# Repurposing screen identifies mebendazole as a clinical candidate to synergise with docetaxel for prostate cancer treatment

**DOI:** 10.1038/s41416-019-0681-5

**Published:** 2019-12-17

**Authors:** Linda K. Rushworth, Kay Hewit, Sophie Munnings-Tomes, Sukrut Somani, Daniel James, Emma Shanks, Christine Dufès, Anne Straube, Rachana Patel, Hing Y. Leung

**Affiliations:** 1 Institute of Cancer Sciences, College of Medical, Veterinary and Life Sciences, University of Glasgow, Bearsden, Glasgow G61 1QH UK; 2 CRUK Beatson Institute, Bearsden, Glasgow G61 1BD UK; 3 Centre for Mechanochemical Cell Biology, University of Warwick, Coventry, CV4 7AL UK; 4 Strathclyde Institute of Pharmacy and Biomedical Sciences, University of Strathclyde, Glasgow, G4 0RE UK

**Keywords:** Prostate cancer, Targeted therapies, Chemotherapy, Cancer therapeutic resistance

## Abstract

**Background:**

Docetaxel chemotherapy in prostate cancer has a modest impact on survival. To date, efforts to develop combination therapies have not translated into new treatments. We sought to develop a novel therapeutic strategy to tackle chemoresistant prostate cancer by enhancing the efficacy of docetaxel.

**Methods:**

We performed a drug-repurposing screen by using murine-derived prostate cancer cell lines driven by clinically relevant genotypes. Cells were treated with docetaxel alone, or in combination with drugs (*n* = 857) from repurposing libraries, with cytotoxicity quantified using High Content Imaging Analysis.

**Results:**

Mebendazole (an anthelmintic drug that inhibits microtubule assembly) was selected as the lead drug and shown to potently synergise docetaxel-mediated cell killing in vitro and in vivo. Dual targeting of the microtubule structure was associated with increased G2/M mitotic block and enhanced cell death. Strikingly, following combined docetaxel and mebendazole treatment, no cells divided correctly, forming multipolar spindles that resulted in aneuploid daughter cells. Liposomes entrapping docetaxel and mebendazole suppressed in vivo prostate tumour growth and extended progression-free survival.

**Conclusions:**

Docetaxel and mebendazole target distinct aspects of the microtubule dynamics, leading to increased apoptosis and reduced tumour growth. Our data support a new concept of combined mebendazole/docetaxel treatment that warrants further clinical evaluation.

## Background

Prostate cancer is the second most common cause of cancer deaths in men in the Western world.^1^ For advanced disease at diagnosis, androgen-deprivation therapy (ADT) remains the main treatment option. Docetaxel is currently the standard cytotoxic regime routinely used to treat metastatic prostate cancer; however, treatment with this drug leads to only a modest increase in median survival.^2,3^ Recent evidence from clinical trials giving hormone-sensitive patients upfront treatment of docetaxel in combination with ADT has demonstrated a robust increase in survival. Subsequently, upfront ADT combination therapy with either chemotherapy or androgen receptor pathway inhibitors has become routinely utilised.^2,4–6^ Despite initial response, however, the majority of tumours relapse within 2–3 years, due to de novo resistance or the emergence of treatment-resistant prostate cancer. In contrast to successes observed in other tumours, including breast cancer,^7^ efforts to develop combination therapies to incorporate docetaxel have not translated into new treatment regimes in prostate cancer.^8^ The use of prostate cancer model systems driven by clinically relevant molecular lesions may identify novel drugs to synergise docetaxel-mediated antitumour effects.

Inactivation of the tumour suppressor PTEN plays a critical role in prostate carcinogenesis. In some tumours, WNT-driven β-catenin activation in combination with PTEN deficiency mediates metastatic castration-resistant prostate cancer (CRPC).^9,10^ Similarly, aberrant cellular signalling associated with combined PTEN and Sprouty2 (SPRY2) inactivation can drive aggressive, treatment-resistant prostate cancer.^11^ Together, these genetic alterations represent a significant portion of prostate tumours. We therefore developed and characterised genetically modified murine models with prostate tumour-specific alterations: (1) *PbCre Ctnnb1*^*(ex3Δ)/+*^ *Pten*^*fl/+*^, with heterozygous deletion of *Ctnnb1* exon 3, leading to constitutive β-catenin activation and heterozygous loss of Pten in the prostatic epithelium; (2) *PbCre Spry2*^*fl/+*^ *Pten*^*fl/fl*^, with heterozygous loss of Spry2 and homozygous loss of Pten.^11^ Prostate cancer cell lines were generated from the *PbCre Ctnnb1*^(ex3Δ)/+^ *Pten*^*fl/*+^ and *PbCre Spry2*^*fl/+*^ *Pten*^*fl/fl*^ tumours, hereafter referred to as CP2 and SP1 cells, respectively. These murine prostate cancer cells form prostate orthografts in immune-compromised nude mice with evidence of metastatic disease, thus recapitulating clinically invasive prostate cancer.

Drug repurposing may fast-track previously unpredicted uses of available drugs. As drugs considered for repurposing have well-characterised pharmacodynamic properties and toxicities, the development time and cost to reach the clinic can be minimised. High-throughput screening of repurposing agents allows the concurrent testing of drug libraries to identify putative candidate therapeutics,^12^ e.g. the use of thalidomide in multiple myeloma.^13^

We conducted a drug-repurposing screen to identify novel therapeutic drugs to combine with docetaxel to treat invasive prostate cancer. The anti-parasitic drug mebendazole was identified as the top candidate to synergise with docetaxel to inhibit cell growth, with suppression of cell cycle progression and increased cell death. This is a result of major disruption to the microtubule network, causing cells to form multipolar spindles and fail to divide correctly.

## Methods

### Methodology for multiple experiments

Details for the following experiments are described in Supplementary Information: cell survival assay, FACS and cell cycle analysis, confocal microscopy and formulation and physicochemical characterisation of liposomes.

### Cell culture

CP2 and SP1 cells were derived from genetically modified mouse prostate cancer models that represent activation of β-catenin and inactivation of Sprouty2 along with the loss of Pten tumour-suppressor protein, respectively.^10,11^ Details of the CP2 (RRID:CVCL_VQ85) and SP1 (RRID:CVCL_VQ86) cell lines have been deposited on the RRID Portal (https://scicrunch.org/resources/). Cells were grown in DMEM supplemented with 10% foetal bovine serum (FBS) and 2 mM l-glutamine. LNCaP and PC3 cells were obtained from American Type Culture Collection and were grown in RPMI supplemented with 10% FBS and 2 mM l-glutamine. RPE1 cell lines stably expressing H2B-RFP, GFP-tubulin or EB3-GFP were maintained in the DMEM/F-12 medium supplemented with 10% FCS, 2.3 g/l sodium bicarbonate, 100 U/ml penicillin, 100 μg/ml streptomycin and 500 µg/ml geneticin. Cell lines were authenticated by LCG standards or in-house using Promega GenePrint 10 Kit. All cell lines used were routinely tested every 6 months for mycoplasma using an in-house MycoAlert™ Mycoplasma Detection Kit (Lonza, Switzerland), according to the manufacturer’s instructions. RPE1 cells were tested monthly for mycoplasma using a MycoSensor PCR Assay Kit (Agilent Technologies, USA).

### Drug libraries

The repurposing libraries used in the screen were the NIH Clinical Collection and NIH Approved Oncology Collection. The Clinical Collection contains 727 small molecules previously used in Phase I–III human clinical trials, and the Oncology Collection contains 130 of the most current FDA-approved anticancer drugs. Libraries were purchased from the NCI Developmental Therapeutic Program's Open Compound Repository, NIH National Cancer Institute (Maryland, USA).

### Repurposing screen

Initial experiments were undertaken to establish a robust screening plan. The optimal seeding densities for the cell lines were ascertained for plating cells in 384-well plates, and dose–response curves for an EC30 of docetaxel were carried out and tested extensively in mock screens. CP2 and SP1 cells were plated out in 384-well plates and treated for 48 h with docetaxel or DMSO in combination with the library drugs. The drugs from the compound libraries were assayed at three different concentrations (0.1, 1 and 10 µM), and all conditions were tested in triplicate. Cells were fixed and stained with DAPI, and the readout was cytotoxicity, quantified by nuclear count using High Content Imaging Analysis (Operetta, Perkin Elmer). Staurosporine (1 µM) was used as a positive control for cytotoxicity. To determine a positive inhibitory test, the mean of the percentage inhibition (PI) in docetaxel-only wells was calculated. The mean PI for the triplicate wells containing library drugs and docetaxel had to be greater than the mean PI of docetaxel-alone wells by 10%. The quality of the screen was assessed primarily using the Z-prime for each plate, and all performed well (average Z-prime value 0.73; max 0.94, min 0.51).

### Synergy assay

Cells were seeded in 96-well opaque white plates and treated the following day with various combinations of drugs in a checkerboard system. Each plate contained 8 × 8-dose matrix blocks with serial twofold dilutions of docetaxel and 1.333-fold dilutions of mebendazole. Additional wells were reserved for untreated and vehicle-treated control wells. Forty-eight hours later, the percentage of growth inhibition was assayed using the CellTiter-Glo Assay (Promega). The combination index (CI) was calculated using CompuSyn software, where <1 indicates synergism, = 1 is an additive effect and >1 indicates antagonism.^14^

### Live-cell imaging

Cells were seeded into glass-bottom dishes (World Precision Instruments), coated with 10 µg/ml fibronectin (Sigma), on the day prior to imaging. In all, 2 nM docetaxel, 200 nM mebendazole, both drugs or DMSO as a control was diluted in growth medium and added to cells immediately before transferring the dish to the microscope-stage incubator (Tokai Hit) heated to 37 °C with 5% CO_2_. RPE1 cells stably expressing EB3-GFP were imaged between 30 and 75 min after drug addition for 100 frames at 1 frame per second using a ×100 NA 1.4 objective on an Olympus personal Deltavision microscope (Applied Precision, LLC) using a GFP filter set (Chroma) and a Coolsnap HQ camera, controlled by Softworx (Applied Precision, LLC). Kymographs were generated from radial lines drawn on the maximum-projected movies and comets traced to measure average microtubule assembly speed from 15 cells for each condition. RPE1 cells stably expressing H2B-RFP or PC3 cells stained with 5 µM SiR-DNA were imaged for > 50 h at 1 frame per 10 min using a ×10 NA 0.3 dry objective on an Olympus personal Deltavision microscope (Applied Precision, LLC) using a mCherry filter set (Chroma) and a Coolsnap HQ camera, controlled by Softworx (Applied Precision, LLC). For each condition, mitotic timing and outcome were analysed in at least 100 (RPE1) or 50 cells (PC3). RPE1 cells stably expressing GFP-tubulin and stained with 5 µM SiR-DNA were imaged for 12 h at 1 frame per 5 min using a ×40  NA 1.2 objective on an Olympus personal Deltavision microscope (Applied Precision, LLC) using GFP and mCherry filter sets (Chroma) and a Coolsnap HQ camera, controlled by Softworx (Applied Precision, LLC).

### In vitro liposome-mediated anti-proliferative activity

Anti-proliferative activity of liposomes encapsulating mebendazole and/or docetaxel was assessed using a standard MTT assay (Sigma-Aldrich). PC3M-Luc-G5 cells were seeded at a density of 5000 cells/well in 96-well plates for 24 h. For LNCaP, 96-well plates were pre-coated with poly-l-lysine solution (6.4 µg/mL, 50 µl per well) overnight. The coating solution was removed, and the plates were washed with PBS (pH 7.4) prior to seeding LNCaP cells at the density of 10,000 cells/well for 24 h. The cells were treated with the liposomal formulation encapsulating docetaxel (0–20 nM), with the liposomal formulation encapsulating mebendazole (0–182.5 nM) or with liposomal formulation encapsulating both drugs at the concentrations mentioned previously for 48 h. The ratio of mebendazole to docetaxel in the formulation was maintained at 9:1. Untreated cells were used as negative controls, and cells treated with 1% Triton X as positive controls. Absorbance was measured at 570 nm using a plate reader.

### In vivo liposome-mediated activity in a subcutaneous xenograft model

The in vivo experiments were carried out in accordance with the UK Home Office regulations (UK Animals (Scientific Procedures) Act 1986) under Project Licence P32E328B7.

PC3M-Luc-G5 cancer cells in exponential growth were subcutaneously injected into both flanks of 7-week-old male immunodeficient BALB/c mice (1 × 10^6^ cells per flank). Mice were ordered from Charles River (UK), housed in randomised groups of five, at 19–23 °C with a 12-h light–dark cycle and were fed a conventional diet (Rat and Mouse Standard Expanded, B&K Universal, UK) with water ad libitum. They were housed in an enriched environment, with igloos, cardboard tubes and chewing sticks. When tumours were palpable and reached a diameter of 5 mm, groups of five mice (thus ten tumours per treatment group) received the following treatments: empty liposomes, untargeted liposomes entrapping docetaxel or mebendazole, untargeted liposomes entrapping docetaxel and mebendazole and transferrin (Tf)-bearing liposomes entrapping docetaxel and mebendazole (for direct comparison with untargeted liposomes entrapping docetaxel and mebendazole). Each mouse received five intravenous tail vein injections (20 mg of docetaxel and 180 mg of mebendazole per kg of body weight per injection) once every 2 days for 10 days (last treatment on day 9). The weight of the animal was measured daily as a surrogate marker of the toxicity of the treatments. One animal from the group treated with transferrin (Tf)-bearing liposomes entrapping docetaxel and mebendazole was removed from the study due to an eye issue (unrelated to treatment). The tumour volume was determined by calliper measurements (volume = d^3^ × π/6), with a defined endpoint of tumour diameter 10 mm. Mice were euthanised humanely by CO_2_ inhalation. The results are expressed as relative tumour volume (rel. Volt_x_ = Volt_x_/Volt_0_) and responses classified analogous to Response Evaluation Criteria in Solid Tumours (RECIST) guidelines. Progressive disease is defined as an increase in relative tumour volume higher than 1.2-fold, stable disease as a relative volume between 0.7 and 1.2 of starting volume and partial response as measurable tumour with a volume reduction of more than 30% (0–0.7).

The therapeutic efficacy of these treatments was also assessed by bioluminescence imaging, using an IVIS Spectrum (Caliper Life Sciences, Hopkinton, MA). Mice bearing subcutaneous PC3M-Luc-G5 tumours were intravenously injected with treatments as described above. On alternate days (Days 1, 3, 5, 7 and 9), animals were intraperitoneally injected with the luciferase substrate d-luciferin (150 mg/kg body weight), then anaesthetised using isoflurane inhalation 10 min before imaging. The light emitted from the bioluminescent tumours was detected for 2 min using Living Image software (Perkin Elmer, Waltham, MA) and displayed as a pseudo-colour overlay onto a greyscale image of the animal. Identical illumination settings were used for acquiring all images.

### Statistics

Data plotting and statistical analyses, including two-way ANOVA, Welch’s *t* test (unpaired, two tailed), Mann–Whitney, Kaplan–Meier survival analysis and log rank (Mantel–Cox), were carried out using GraphPad Prism 7. Graphs are shown as mean ± standard deviation (SD) or standard error of the mean (SEM) with individual points shown.

## Results

### A repurposing screen identifies drugs that increase docetaxel efficacy

We performed a drug-repurposing screen in cell lines derived from aggressive tumours developed in *Pbcre Ctnnb1*^*(ex3Δ)/+*^ *Pten*^*fl/+*^ and *PbCre Spry2*^*fl/+*^ *Pten*^*fl/fl*^ mice (CP2 and SP1 cells, respectively). These tumours resulted from genetic alterations to important signalling pathways in prostate cancer, making them clinically highly relevant.^10,11^ CP2 and SP1 cells signify carcinogenesis driven by WNT and Ras-MAPK pathways, respectively (Supplementary Fig. S1A). The screen workflow is summarised in Supplementary Fig. S1B, C. Briefly, CP2 and SP1 cells were treated with docetaxel (at predicted EC30: CP2, 7 nM; SP1, 5 nM, respectively; Supplementary Fig. S1D) or DMSO in combination with drugs from two repurposing libraries—NIH Clinical Collection and NIH Approved Oncology Collection. The drugs were assayed at three different concentrations (0.1, 1 and 10 µM), and the readout was cytotoxicity quantified by nuclear count. The percentage growth inhibition relative to the DMSO control for each drug was calculated. The drug window that would support combination therapy was defined as a ≥ 10% increase in growth inhibition when combined with docetaxel.

A total of 820 unique drugs were tested in the primary screen, with some overlap (*n* = 37) between the two libraries. Analysis of the percentage growth inhibition of the 37 duplicated drugs showed that these caused similar effects, providing a convenient internal quality control (Supplementary Fig. S1E). One of the drugs was docetaxel itself, which at 0.1 µM resulted in 80–90% inhibition in both cell lines as expected (Supplementary Fig. S1F). Other well-known anticancer drugs were also effective as single agents in both cell lines at the lowest concentration tested. Most of the drugs however had little or no effect on either cell line at 0.1 µM (Supplementary Fig. S1F).

**Fig. 1:**
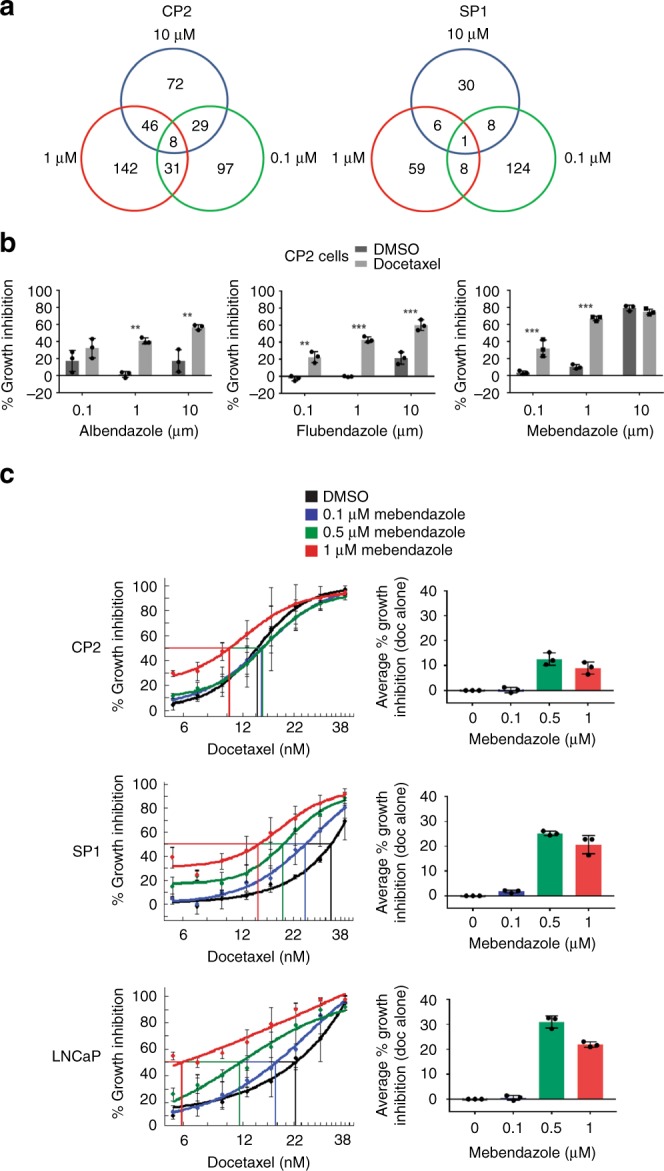
Drug-repurposing screen identifies drugs that increase docetaxel efficacy. **a** Venn diagram of screen results. The percentage growth inhibition for each drug in combination with docetaxel relative to the DMSO control was calculated. The number of drugs with a ≥ 10% increase is shown for each concentration. **b** CP2 cells were treated with the indicated anthelmintics, combined with either docetaxel or DMSO. The percentage growth inhibition for each drug in combination with docetaxel relative to the DMSO control was calculated. *n* = 3 (technical replicates), mean values ± SD are shown, analysed by two-way ANOVA with Sidak’s multiple comparisons test, *p < 0.01, **p < 0.001, ***p < 0.0001. **c** CP2, SP1 and LNCaP cells were treated with mebendazole at the indicated concentrations or DMSO control, in combination with docetaxel at a range of concentrations for 48 h. Cells were fixed, stained with DAPI and cytotoxicity quantified by nuclear count using High Content Imaging Analysis (Operetta, Perkin Elmer). The percentage growth inhibition relative to the DMSO control for each docetaxel concentration (left panels), and the percentage cell survival after treatment with the target drugs alone (right panels) were calculated. *n* = 3 (technical replicates), mean values ± SD are shown.

Drugs exhibiting ≥ 10% increase in growth inhibition when combined with docetaxel were identified (Fig. 1a; Supplementary S1G, H). Relatively fewer hits were identified in SP1 cells (Fig. 1a; Supplementary S1I; Table S1), which likely reflects the actual percentage inhibition achieved by docetaxel alone in the screen, at 17 and 42%, respectively for SP1 and CP2 cells. Among the top hits for CP2 cells were the anthelmintic family of drugs. This includes albendazole, flubendazole and mebendazole, which showed additional effects on cell survival with docetaxel at all concentrations tested (except albendazole at 0.1 µM and mebendazole at 10 µM; Fig. 1b). These drugs are anti-parasitics used to expel worms by suppressing microtubule assembly. Importantly, mebendazole was the only drug that increased the percentage growth inhibition in both cell lines, albeit to a lesser extent in SP1 cells (Supplementary Fig. S1J), suggesting that this family of drugs may provide a combinatorial docetaxel effect, irrespective of genetic alteration. Two of these drugs, mebendazole and albendazole, were selected for validation along with other lead candidates, dabrafenib, honokiol and nobiletin (Supplementary Table S2 provides the current uses of these drugs, as well as detailing previous cancer studies and associations with prostate cancer). All of these drugs have shown previous efficacy in multiple cancer models both in vitro and in vivo. To validate these drugs, incremental doses of docetaxel (*n* = 8, 5.3–40 nM) were combined with target compounds in CP2 and SP1 cells, and human LNCaP prostate cancer cells. Among the five hits evaluated (Fig. 1c; Supplementary Fig. S2A), mebendazole showed the greatest combinatorial effect with docetaxel in a dose-dependent manner, particularly in SP1 and LNCaP cells (Fig. 1c). Mebendazole was therefore tested in additional survival assays.

**Fig. 2:**
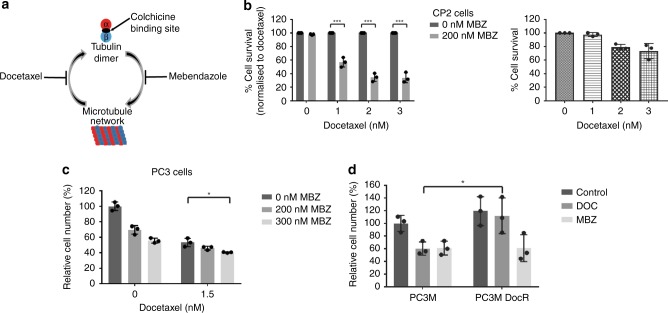
Combining docetaxel and mebendazole increases growth inhibition. **a** Schematic of the mode of action of docetaxel and mebendazole on microtubule networks. Mebendazole binds to the colchicine- binding site, while docetaxel stabilises the microtubule structure by binding to its inner surface. **b** CP2 cells were treated with different drug combinations (MBZ = mebendazole) and the IncuCyte (Essen Bioscience) used to calculate the decrease in percentage cell survival (left panel). Percentage cell survival was normalised to docetaxel treatment. *n* = 3 (independent experiments), mean values ± SD are shown, analysed by two-way ANOVA with Sidak’s multiple comparisons test, ***p < 0.0001. (The effects of docetaxel alone are shown in the right panel.) **c** PC3 cells were treated with the indicated drugs and analysed for changes in growth inhibition by cell counting. *n* = 3 (independent experiments), mean values ± SD are shown, analysed by two-way ANOVA with Sidak’s multiple comparisons test, *p < 0.01. **d** PC3M and docetaxel-resistant PC3M cells (PC3M DocR) were seeded in normal media 24 h before being treated with the indicated drugs and analysed for changes in growth inhibition by cell counting. *n* = 3 (independent experiments), mean values ± SD are shown, analysed by two-way ANOVA with Sidak’s multiple comparisons test, *p < 0.05.

Docetaxel inhibits the depolymerisation of microtubules by binding to the inner surface of β-tubulin, while mebendazole inhibits the polymerisation of microtubules by binding to tubulin dimers at the colchicine site (Fig. 2a).^15^ We therefore hypothesised that these drugs may work together to synergistically affect the microtubule dynamics. Combining mebendazole with docetaxel decreased cell survival in both CP2 (Fig. 2b) and SP1 cells (Supplementary Fig. S2B). Similar to the observed effects in human LNCaP cells (Fig. 1c), combining docetaxel and mebendazole showed enhanced suppression of PC3 cell survival (Fig. 2c). As a single agent, mebendazole was also able to decrease cell survival in docetaxel-resistant cells (PC3M DocR) to a similar extent as the parental PC3M cells, suggesting that mebendazole may have the potential to be used as treatment for docetaxel-resistant prostate cancers (Fig. 2d).

### Docetaxel and mebendazole work together to increase apoptosis

**Fig. 3:**
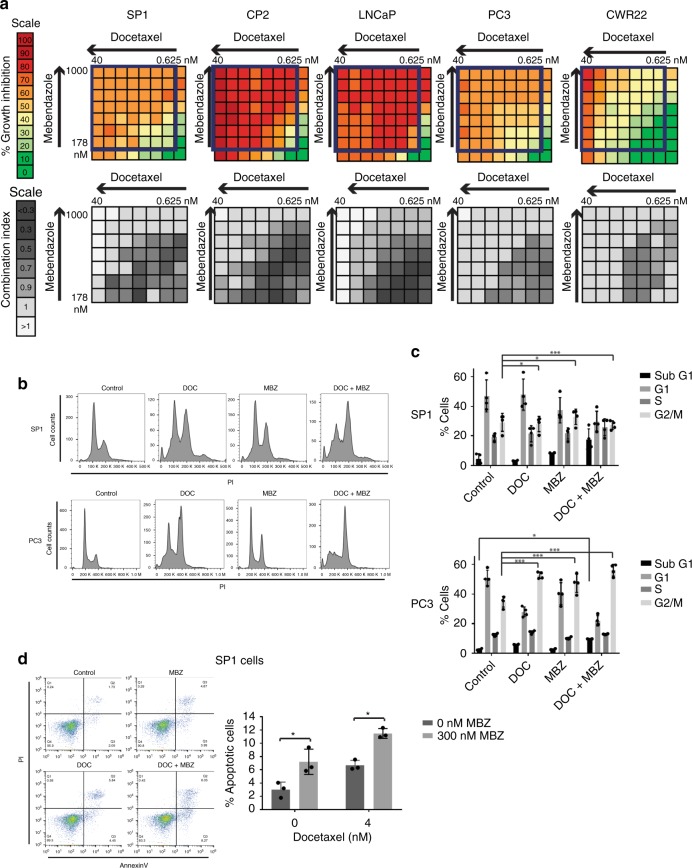
Docetaxel and mebendazole work in a synergistic manner to increase apoptosis associated with G2/M block. **a** Cells were treated with docetaxel and mebendazole at the indicated concentrations for 48 h. Each plate contained 8 × 8-dose matrix blocks with serial twofold dilutions of docetaxel and 1.333-fold dilutions of mebendazole. Percentage growth inhibition was calculated for each drug combination using the CellTiter-Glo Assay (Promega). The combination index (CI) was calculated using CompuSyn software, where < 1 indicates synergism,  = 1 is an additive effect and > 1 indicates antagonism. The percentage and colour codes for growth inhibition and CI scales are shown on the left. **b** SP1 (upper panel) and PC3 (lower panel) cells were synchronised using a double-thymidine block before being drug treated, stained with propidium iodide (PI) and analysed using flow cytometry. Both drugs individually cause a G2/M arrest, and the block is further increased when the cells are treated with both drugs. *n* = 3, a representative image is shown. **c** Percentages of cells in each phase of the cell cycle were calculated and plotted. *n* = 3, mean values ± SD are shown, analysed by two-way ANOVA with Tukey’s multiple comparisons test, *p < 0.05, ***p < 0.0001. **d** Cells were treated with different drug combinations, stained with annexin V and propidium iodide and analysed by flow cytometry (left panel, a representative image is shown). The percentage of cells stained with annexin V is shown in the right panel. *n* = 3 (independent experiments), mean values ± SD are shown, analysed by two-way ANOVA with Sidak’s multiple comparisons test, *p < 0.01.

We wanted to ascertain if the use of mebendazole and docetaxel simultaneously resulted in synergistic cell growth inhibition. The combination index (CI) determines whether the combination is synergistic (< 1), additive (= 1) or antagonistic (> 1).^14^ Using a luminescence proliferation assay, we calculated the percentage growth inhibition and CI for docetaxel and mebendazole using dose curves in a checkerboard manner (Fig. 3a). In the mouse cell lines, as well as the human cell lines LNCaP, PC3 and CWR22, synergy between the drugs was seen, particularly at lower concentrations. For example, for 1.25 nM docetaxel and 237 nM mebendazole, the CI values for SP1, CP2, LNCaP, PC3 and CWR22 cells are 0.48, 0.71, 0.39, 0.78 and 0.80, respectively, confirming that the drugs are synergistic at these concentrations in all the cell lines tested.

Cell cycle analysis of SP1 and PC3 cells revealed enhanced G2/M and sub-G1 fractions following combined docetaxel and mebendazole treatment (Fig. 3b, c). Furthermore, apoptosis in SP1 and CP2 cells was induced following combined treatment (Fig. 3d; Supplementary Fig. S3A). Untransformed RWPE human prostate epithelial cells were less responsive to the two drugs in isolation than cancer cells (Supplementary Table S3), although a degree of synergism to combined treatment was observed (Supplementary Fig. S3B). Since mebendazole binds to the colchicine-binding site of the tubulin dimer, colchicine was also studied and shown to enhance docetaxel-induced cell death, providing mechanistic support for therapeutic synergism arising from distinct interactions within the microtubule structure (Supplementary Fig. S3C).

### Combining docetaxel and mebendazole perturbs microtubule dynamics and leads to aberrant mitosis

**Fig. 4:**
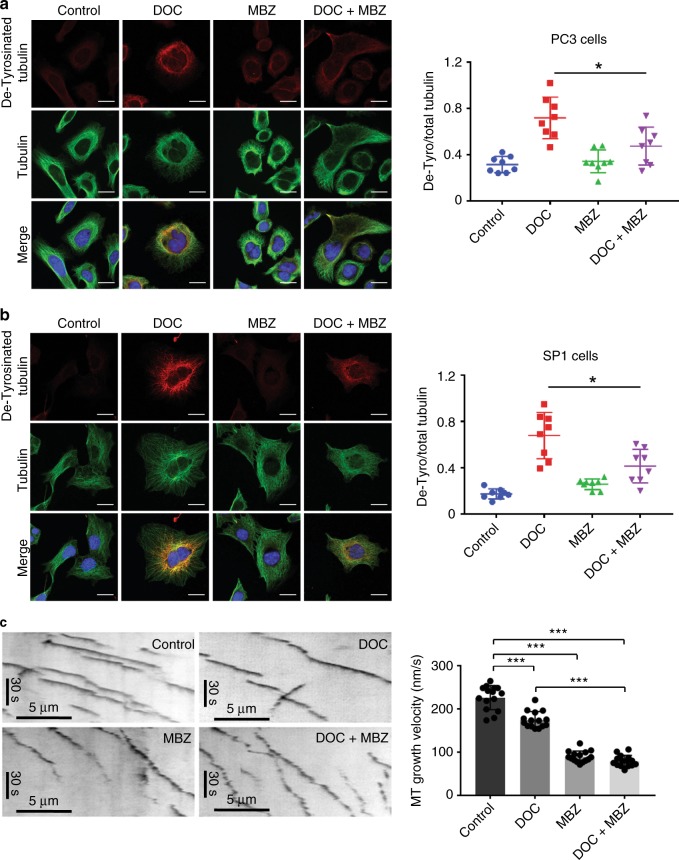
Combining docetaxel and mebendazole perturbs microtubule dynamics by reducing microtubule assembly speed. **a** PC3 and **b** SP1 cells were seeded on coverslips and treated with the indicated drugs for 24 h before methanol fixation. Cells were stained for detyrosinated tubulin (red) and total tubulin (green) and visualised using confocal microscopy (scale bar is 20 µm). For each cell line, fluorescence intensity of detyrosinated tubulin in individual cells was measured using ImageJ and normalised to total tubulin intensity. *n* = 8 (from two independent experiments), mean values ± SD are shown, analysed by one-way ANOVA with Tukey’s multiple comparisons test, *p < 0.01. **c** Kymographs of EB3-GFP comets in RPE1 cells treated with 2 nM docetaxel and/or 200 nM mebendazole. Left panels show representative images. The right panel shows microtubule assembly speeds measured from 15 cells for each condition as indicated. Data were pooled from two independent experiments. *n* = 15, mean values ± SD are shown, analysed by one-way ANOVA with Tukey’s multiple comparisons test, ***p < 0.0001.

Given that the primary target of both drugs is tubulin, we wanted to look more closely at the microtubule network. Tubulin can be modified in various ways, including detyrosination, which accumulates in stable microtubules.^16^ We performed confocal microscopy in PC3 and SP1 cells using antibodies against detyrosinated tubulin to study the effect of the drug combinations on microtubule stability (Fig. 4a, b). Docetaxel-treated cells showed significantly elevated levels of detyrosinated tubulin. The addition of mebendazole interestingly significantly reduced docetaxel-mediated accumulation of detyrosinated tubulin. These data suggest that the two drugs in isolation have distinct effects on microtubule stability. Microtubule dynamics were then studied in RPE1 (hTERT retinal pigment epithelium 1-positive) cells stably expressing a GFP-tagged end-binding protein EB3 to visualise microtubule growing ends. Treatment with docetaxel reduced growth speed slightly compared with the control cells, while mebendazole decreased growth speed to a much greater extent (Fig. 4c). Combining the drugs did not significantly further reduce microtubule growth compared with mebendazole alone.

**Fig. 5:**
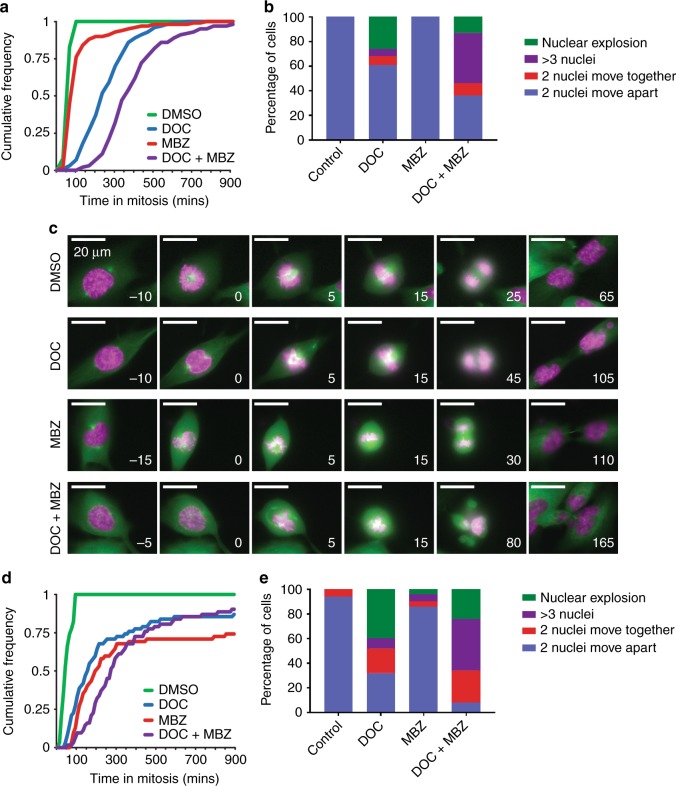
Combination treatment extends mitotic delay and leads to aberrant mitosis. **a** Cumulative frequency plot showing time spent in mitosis (from chromosome condensation to anaphase) for RPE1 H2B-RFP cells treated with the indicated drugs (2 nM docetaxel, 200 nM mebendazole). One hundred cells per condition were analysed. The median distributions are significantly different from each other at 95% confidence (Mann–Whitney U test). **b** RPE1 H2B-RFP cells treated with the indicated drugs (100 cells per condition) were analysed for mitotic outcome. The percentage of daughter cells in each of four categories was calculated. **c** Mitotic progression of RPE1 GFP-tubulin cells stained with 0.5 µM SiR-DNA (pink) and treated with the indicated drugs. Time is shown in minutes relative to nuclear envelope breakdown (time shown at the right bottom corner of each image; range −5 to + 165 min; treatment at 0 min). **d** Cumulative frequency plot showing time spent in the mitosis (from chromosome condensation to anaphase) for PC3 cells treated with the indicated drugs (2 nM docetaxel, 200 nM mebendazole) and stained with 0.5 µM SiR-DNA. Sixty-two cells per condition were analysed. The median distributions are significantly different from each other at 95% confidence (Mann–Whitney U test). **e** PC3 cells treated with the indicated drugs (50 cells per condition) and stained with SiR-DNA were analysed for mitotic outcome. The percentage of daughter cells in each of four categories was calculated.

The use of histone H2B as a nuclear marker helps to determine the mitotic timing, defined as time from chromosome condensation to anaphase (Fig. 5a). Studying RPE1 cells expressing H2B-RFP, the median time spent in mitosis was significantly increased with combined treatment (Fig. 5a). Analysis of mitotic outcome revealed that docetaxel treatment had a large effect on the number of daughter cells with abnormal, fragmented nuclei (Fig. 5b). Perhaps surprisingly given its profound effect on microtubule growth speed in RPE1 cells, mebendazole as a single agent had little effect on mitotic outcome. All cells treated with mebendazole alone successfully divided, compared with only 61% of docetaxel-treated cells. Combined treatment had a profound effect on spindle formation, resulting in a severe increase in multipolar divisions, in addition to a high prevalence of fragmented nuclei. Strikingly, none of the combination-treated cells divided properly, so even though 36% of cells had two nuclei moving apart, in no case were both nuclei normal. (Supplementary Fig. S4 provides examples of the classes of mitotic outcome.) GFP-tubulin-expressing RPE1 cells allowed more detailed investigation into mitotic spindle formation using time-lapse microscopy (Fig. 5c). DMSO-treated cells showed distinct poles with proper formation of the mitotic spindle and metaphase plate, while combination therapy produced multipolar spindles with failure to congress chromosomes, resulting in three abnormal daughter cells. Similarly, combination treatment of PC3 cells also increased the median time spent in mitosis and the percentage of daughter cells with aberrant nuclei (Fig. 5d, e). Interestingly, the effect on PC3 cells was even more pronounced than that on the RPE1 cells, with 92% of cells failing to undergo mitosis to produce two nuclei. This failure to divide correctly is consistent with the observed increase in G2/M block and cell death (Fig. 3b–d; Supplementary S3A).

### In vitro and in vivo evaluation with liposome-mediated combination treatment

In order to test the drug combination as a proof-of-concept experiment, we employed liposomes encapsulating docetaxel, mebendazole or both drugs. In addition, transferrin (Tf)-bearing liposomes entrapping both docetaxel and mebendazole were prepared for head-to-head comparison with untargeted liposomes encapsulating both drugs. Transferrin targets the liposomes to cancer cells expressing increased levels of the transferrin receptor.^17–19^ The ratio of mebendazole to docetaxel in the combination liposomes was stable at 9:1, and the liposomal formulations were all <150 nm in size (<200 nm is essential for the enhanced permeation and retention (EPR) effect) (Supplementary Table S4).

To assess the anti-proliferative activity of the liposomes in vitro, liposomes encapsulating mebendazole and/or docetaxel were assessed using an MTT assay in PC3M-Luc-G5 and LNCaP cells. Cells were treated with liposomal formulations encapsulating docetaxel (0–20 nM), mebendazole (0–182.5 nM) or both drugs at the same concentrations (Supplementary Fig. S5). The ratio of mebendazole to docetaxel in the formulation was maintained at 9:1. Tf-targeted and untargeted liposomes containing combined docetaxel and mebendazole were shown to have comparable in vitro efficacy, with transferrin targeting achieving higher efficacy in suppressing cell survival, particularly in LNCaP cells treated with lower combined drug doses.

In vivo efficacy was evaluated in a subcutaneous PC3M-Luc-G5 xenograft model.^20^ Briefly, PC3M-Luc-G5 cells were subcutaneously injected into both flanks of male immunodeficient BALB/c mice. Animals were treated intravenously every 2 days for 9 days (20 and 180 mg/kg of body weight per injection for docetaxel and mebendazole, respectively) with empty liposomes, untargeted liposomes entrapping docetaxel or mebendazole, untargeted liposomes entrapping docetaxel and mebendazole or Tf-bearing liposomes entrapping docetaxel and mebendazole. Experimental mice injected with Tf-bearing liposomes containing both docetaxel and mebendazole are to be directly compared with mice treated with untargeted liposomes entrapping both drugs in order to investigate the in vivo impact of transferrin-mediated delivery of the liposome complex.

**Fig. 6:**
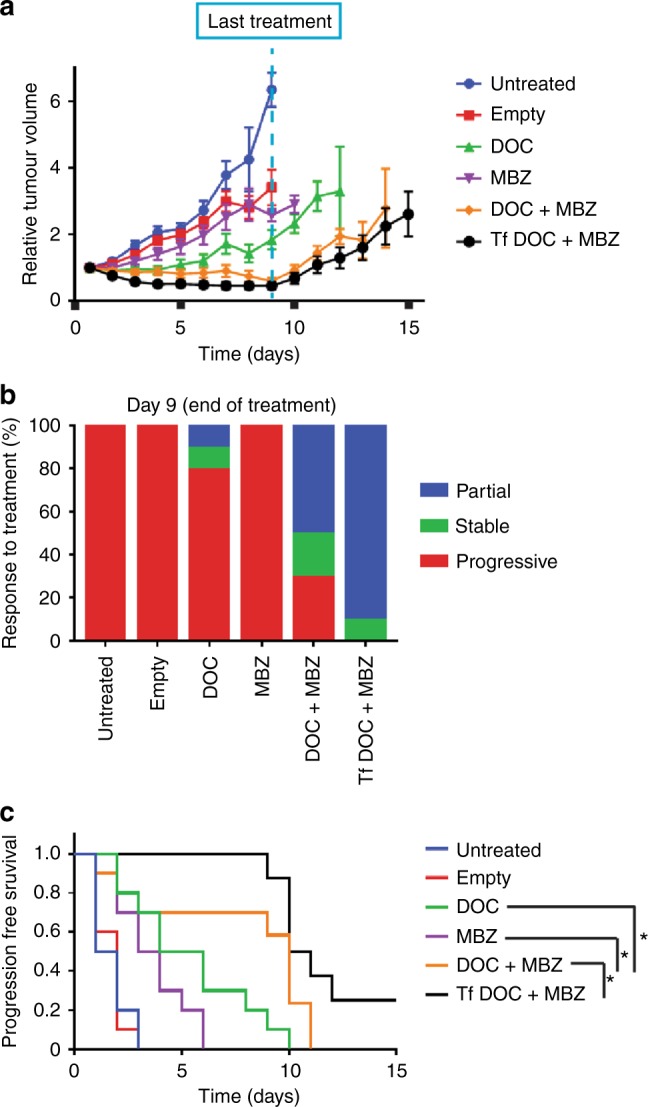
Tumour growth studies in a PC3M-Luc-G5 murine model. **a** PC3M-Luc-G5 cells were subcutaneously injected into male immunodeficient BALB/c mice, and when tumours were palpable and reached a diameter of 5 mm, the animals were randomised to receive intravenous administration of untargeted liposomes entrapping docetaxel and mebendazole (DOC + MBZ), docetaxel only (DOC) or mebendazole only (MBZ), along with control mice that were either untreated or injected with empty liposomes. In addition, a group of mice were injected with transferrin (Tf)-bearing liposomes entrapping DOC + MBZ for a focused comparison with untargeted liposomes entrapping DOC + MBZ. *n* = 10 (except for Tf DOC + MBZ, *n* = 8), mean values ± SEM are shown. **b** Overall tumour (*n* = 10) response at the end of treatment, classified in accordance with the Response Evaluation Criteria in Solid Tumours (RECIST), where progressive disease = increase in relative tumour volume higher than 1.2-fold, stable disease = relative volume between 0.7 and 1.2 and partial response = measurable tumour with a volume reduction of more than 30% (0–0.70). **c** Progression-free survival curves. The *y* axis gives the proportion of tumours that are progressing over time. Progression-free survival was defined as < 20% increase in tumour volume from day 1. *n* = 10 (except for Tf DOC + MBZ, *n* = 8), analysed by log-rank (Mantel–Cox) test, *p < 0.05.

Treatment with untargeted and Tf-bearing liposomes entrapping docetaxel and mebendazole abolished tumour growth (Fig. 6a). During treatment (first 9 days), tumours regressed with dual-drug (untargeted and targeted) treatment, contrasting to continued growth during singleton treatments (Fig. 6a). At the end of treatment, tumours were categorised as progressive, stable or partially regressed (according to RECIST guidelines). Tumours treated with untargeted and Tf-bearing liposomes entrapping both drugs were classified as partially regressed at 50% and 90%, respectively, compared with the progressive nature of the majority of tumours treated with either liposomes containing singleton drug or empty liposomes (Fig. 6b). Bioluminescence imaging of PC3M-Luc-G5 tumours also showed superior efficacy with dual treatment, which resulted in reduced luminescence signals (Supplementary Fig. S6A). No significant variations of animal body weight (Supplementary Fig. S6B) or apparent toxicities were observed.

We defined progression-free survival as < 20% increase from starting tumour volume (day 1). Mice treated with untargeted liposomes containing both docetaxel and mebendazole had extended progression-free survival when compared with mice treated with untargeted liposomes containing docetaxel alone (*p* = 0.038), mebendazole alone (*p* = 0.007) or no active drug (empty liposomes) (*p* = 0.0008), as well as with untreated mice (*p* = 0.0007) (Fig. 6c). The median progression-free survival was extended by 5 and 6.5 days compared with docetaxel and mebendazole alone, respectively (Supplementary Table S5). Furthermore, in a direct comparison of untargeted and Tf-targeted liposomes entrapping both drugs, the delivery of the loaded liposomes via transferrin further extended the duration of progression-free survival in treated animals (*p* = 0.048).

## Discussion

Drug repurposing may have a transformative impact on precision medicine,^21^ fast-tracking novel treatment strategy using agents that would otherwise not be implicated by conventional approaches. Strategies for drug repurposing include a chemogenomic library in phenotypic screen,^22^ in silico computational drug repositioning,^23^ pairwise combinatorial screen of drugs against molecular targets of interest^24^ and novel in vivo model systems such as zebrafish.^25^ The use of cytotoxic docetaxel chemotherapy is well established in metastatic prostate cancer, but only results in a modest survival benefit. Cabazitaxel is approved by the FDA as a second-line chemotherapy treatment; however, again the survival benefit is only moderate, leaving an unmet need for improved therapies.^26^ Our drug-repurposing screen was designed to identify drugs that could synergise with docetaxel and bring forward as clinical candidate(s) as part of a combination therapeutics to treat advanced prostate cancer. To date, none of the earlier efforts to identify a synergiser for docetaxel in prostate cancer using a candidate approach have been confirmed in patients.^27,28^ We hypothesised that a drug-repurposing screen using murine prostate cancer cells harbouring genetic lesions implicated in treatment resistance may identify authentic hits that would not otherwise have been revealed using long-term cultured human cancer cell lines. The genotypes driving carcinogenesis in CP2 and SP1 cells represent a significant proportion (> 60%) of invasive prostate cancer (Supplementary Fig. S1A).

The use of docetaxel in a screen was technically challenging as it has a largely ‘all or nothing’ effect on cells, resulting in a steep dose curve. It was difficult to accurately achieve an EC30 for docetaxel in the screen; we observed a lower-than-desired percentage inhibition in the SP1 cells, likely the main reason for fewer hits in these cells. Nonetheless, the screen revealed a number of interesting drugs that could potentially work in combination with docetaxel. The drug family that showed the greatest synergy with docetaxel was the anthelmintic family. Mebendazole has been studied in isolation in several cancer types (adrenocortical carcinoma, melanoma, HNSCC and colon,^29–32^ including an ongoing Phase I study in paediatric brain tumours, NCT02644291). As an anti-parasitic drug, the dosage of mebendazole varies according to specific infections. Pinworms only require a single treatment, while treatment for echinococcosis may require a prolonged course. Side effects can include abdominal pain and diarrhoea. In general, mebendazole is very well tolerated, making it an excellent candidate for repurposing.^33^ Based on the combination index analysis, mebendazole convincingly synergised docetaxel-mediated growth inhibition in vitro, with greatest synergism observed at low concentrations, suggesting an appealing therapeutic window. Our data were in keeping with previous studies examining taxanes combined with vinca alkaloids (agents that disrupt microtubule function by inhibiting depolymerisation), which also demonstrated synergism at low doses, and additive or antagonistic effects at higher doses.^34,35^ Mechanistically, combined docetaxel and mebendazole reduced microtubule assembly and drastically impaired microtubule dynamics, resulting in aberrant cell division with frequent formation of multipolar spindles, aneuploid daughter cells or arrest in prometaphase.

Mebendazole alone has a dramatic effect on microtubule assembly, yet mitosis was largely unaffected (unlike docetaxel that had the opposite effect). This perhaps suggests that supressing microtubule assembly alone is not enough to perturb mitosis, and that the synergy of the two drugs can have more impact on mitosis due to reduced microtubule turnover. Docetaxel reduces shrinkage of microtubules, and from the mitotic progression experiments, it is clear that growing slower does not have large effects on mitotic outcome, but being less able to shrink microtubules produced a significant problem for mitosis. Like mebendazole, colchicine did not initiate aneuploidy in A549 cells^36^ but synergised with docetaxel, albeit less potently, highlighting the impact of dual-targeting microtubules. Importantly, cells that are resistant to docetaxel were still susceptible to growth inhibition by mebendazole. Our observations are in keeping with a recent study on breast cancer cells, which demonstrated that docetaxel-resistant cells were more sensitive to treatment with colchicine (and other colchicine-site-binding agents) than their parental cells, while other microtubule-targeting agents binding at other sites were less effective.^37^ Taken together, our data show that the combination of docetaxel and mebendazole could be used to treat prostate cancer and potentially combat the issue of docetaxel resistance. Non-tumorigenic RWPE prostatic epithelial cells were less responsive to docetaxel and/or mebendazole treatment (Supplementary Table S3 and Supplementary Fig. S3B), suggesting a potential favourable therapeutic window.

AR pathway-independent forms of CRPC are increasing in incidence with the advent of potent AR-targeting therapies, such as enzalutamide and abiraterone. Besides taxane chemotherapy, radium-223 can be used to treat AR-independent CRPC, particularly for patients with bone metastases.^38,39^ For in vivo synergism studies, we used PC3M cells that represent an AR-independent CRPC form. As a proof-of-concept study, we originally undertook an experiment where CD-1 nude mice were injected with PC3M cells in the anterior prostate and treated with docetaxel (given intraperitoneally) and/or mebendazole (given orally). We found that the mice treated with docetaxel had severe abdominal distension, resulting in removal from the study before the end of treatment.^40^ To counteract this docetaxel-associated toxicity, liposomes were employed to deliver docetaxel and mebendazole to the tumour. Using this approach, we observed enhanced in vivo efficacy with combined treatment. In addition, we found that the efficacy could be further improved by targeting the liposomes to tumour cells using transferrin.^41^ Liposome-mediated drug delivery avoided the docetaxel-associated toxicities that we previously observed. Future clinical study does not necessarily involve liposomes as both drugs are extensively used clinically, and the relative dosing of the two agents can be tested easily. Importantly, the abdominal distension that we observed in mice treated with unencapsulated docetaxel is not often observed in the clinic.

Future clinical studies of combined docetaxel and mebendazole treatment in prostate cancer patients will incorporate careful scheduling to determine the optimal treatment scheme to produce synergism and at the same time minimise systemic toxicity. As the relative dose of docetaxel required will be low, it may be that combined treatment may produce enhanced efficacy without increased toxicities. Finally, colchicine, commonly used for gout, is not known to increase treatment toxicity of docetaxel. Collectively, our repurposing screen using prostate cancer cells derived from genetically engineered mouse models identified mebendazole as a clinical candidate to be combined with docetaxel for synergistic cancer cell killing.

## Supplementary information

Supplementary Information

## Data Availability

Supplementary methods and figures can be found on the *British Journal of Cancer’s* website. Full details of the drug-repurposing data set produced and analysed during this study are available from the corresponding author on reasonable request.
