# Supplementary material for: Repurposing screen identifies mebendazole as a clinical candidate to synergise with docetaxel for prostate cancer treatment
Source: Br J Cancer. 2019 Dec 17;122(4):517–27. doi: 10.1038/s41416-019-0681-5 (PMC7028732; doi:10.1038/s41416-019-0681-5)
Supplement: Supplementary file 1 — Supplementary Information [file 41416_2019_681_MOESM1_ESM.pdf]

Repurposing screen identifies mebendazole as a clinical candidate to synergise with docetaxel for prostate cancer treatment

Supplementary Methods

Cell Survival Assay

Cells were seeded in 96-well plates and treated with various drug combinations and concentrations the following day. The Incucyte Zoom (Essen Bioscience) was used to measure percentage cell confluence every two hours for a total of 96 hours, and data were analysed using Incucyte software.

FACS and Cell Cycle Analysis

Cells were treated with various drug combination concentrations for 24 hours, then all cells (floating and attached) were collected. Cells were washed in PBS and stained with annexin V and propidium iodide (PI) using the FITC Annexin V Apoptosis Detection Kit I (BD Biosciences) according to the manufacturer's instructions. Cells were assayed using the Attune NxT Flow Cytometer (Thermo Fisher Scientific). For cell cycle analysis, cells were double thymidine blocked (2 mM thymidine) before releasing and treating with drugs. Cells were fixed in 70% ethanol and incubated for 30 min at 4°C before washing twice in PBS. Fixed cells were treated with RNase and PI was used to stain cells before analysis.

Confocal Microscopy

PC3 and SP1 cells were treated with various drug combination concentrations for 24 hours, then fixed using ice cold 100% methanol for 10 minutes. Cells were incubated in

blocking buffer (0.5% BSA in PBS) for 15 minutes before adding primary antibodies (anti-de-tyrosinated tubulin, Merck, 1:500; anti-tubulin yl1/2, Abcam, 1:1000) in blocking buffer for one hour, followed by secondary antibody for a further hour. Coverslips were mounted using Diamond Prolong with DAPI (Thermo Fisher Scientific). Cells were imaged using a 60x oil objective on a Nikon A1R confocal microscope (Nikon Instruments Europe B.V.).

Formulation and physicochemical characterization of Liposomes

Liposomes encapsulating docetaxel, mebendazole, or both were formulated using a sonication method. Briefly, a mixture of 1,2-dioleoyl-sn-glycero-3-phosphocholine (DOPC) (20 mg), cholesterol (4.8 mg) and 1,2-distearoyl-sn-glycero-3-phosphoethanolamine-N-[amino(polyethylene glycol)-2000] (DSPE-PEG2000) / DSPE-PEG2000-maleimide (3.6 mg) in PBS (2 ml) were stirred at 60° C for 1 hour, before being sonicated with a Soniprep 150 (Measuring and Scientific (UK) Ltd) for 10 minutes (5 cycles of 2 minutes each). Docetaxel (250 nmoles) and/or mebendazole (5000 nmoles) solutions (in DMSO) were added during the first cycle of sonication. The total lipid concentration in all the formulations was 10 mM. Unentrapped drugs and solvent were removed from liposomes using Vivaspin 6 centrifugal concentrator with a molecular weight cut-off at 100 000 Daltons at 4500 g for 15 minutes. For further purification, the liposomal formulation was filtered through a 0.8 µm surfactant free cellulose acetate syringe filter. The encapsulation efficiency of mebendazole and/or docetaxel in the liposomal formulation was measured using the UV visible spectrophotometer at 229 nm and 312 nm respectively after disruption of liposomes using isopropanol. The encapsulation efficiency was more than 80% for docetaxel, and 42 – 45% for mebendazole, both for each drug alone and in combination. Linear standard curves of mebendazole (0 – 20

μg/mL) and docetaxel (0 – 2 μg/mL) were plotted at the previously mentioned UV Visible wavelengths.

For formulation of transferrin-targeted liposomes, transferrin was thiolated to be able to react with the thiol-reactive maleimide group of cholesterol-PEG-maleimide. To do so, 10 mg of transferrin were dissolved in 1 ml of 50 mM sodium phosphate and 150 mM sodium chloride buffer (pH 8), and reacted with 10-fold molar excess of 2-iminothiolane (Traut's reagent, 85 µl, 2 mg/ml in distilled water) at 25 °C for 1 hour. The thiolated transferrin was then isolated from unreacted Traut's reagent using Vivaspin 6 centrifuge tubes with a molecular weight cut-off of 5000 Daltons (Sartorius Ltd., Epsom, UK), after centrifugation at 10500 g for 15 min at 20 °C (Hermle Z323K centrifuge, Wehingen, Germany). The freshly synthesized thiolated transferrin (Tf) was immediately conjugated to the control liposomes under continuous stirring at 25 °C for 2 hours. Free drugs and/or unreacted Tf were removed from both Tf-bearing and control liposomes using Vivaspin 6 centrifuge tubes with a molecular weight cut-off of 100 000 Daltons (Sartorius Ltd., Epsom, UK) by centrifugation at 4500 g for 30 min at 20 °C. Size and zeta potential of the vesicles were respectively determined by photon correlation spectroscopy and laser Doppler electrophoresis on a Zetasizer Nano-ZS (Malvern Instruments, Malvern, UK).

Supplementary Figure Legends

Figure S1. (A) Illustration of the involvement of WNT and Sprouty2 mediated signalling in prostate cancer (signified by combined alterations in *CTNNB1* and *Sprouty 2* respectively along with loss of *PTEN* function). PTEN, SPRY2 and APC act as tumour suppressors by inhibiting the oncogenic signalling pathways. Bar charts show incidence of genomic alterations (amplifications, copy number gain and mutations) in prostate cancer datasets obtained from cBioPortal Genomics platform. (B) Schematic of screen workflow. (C) Schematic of screen and downstream workflow. (D) Seeding density and docetaxel concentration optimisation. The indicated number of CP2 cells (top panel) and SP1 cells (bottom panel) were seeded out and treated the following day with the docetaxel concentrations shown for 48 hours. The percentage growth inhibition is shown. The final seeding densities and docetaxel concentrations selected for the final screen are highlighted with a * (signifying a predicted EC30). n = 3 (technical replicates), mean values ± SD are shown. (E) Growth inhibition analysis of 37 shared drugs in the two libraries showing highly consistent data. Docetaxel is shown in green. (F) Scatter plot showing the effectiveness (percentage growth inhibition) of all library drugs as stand alone agents at 0.1 µM in SP1 and CP2 cells (Y and X axes respectively). Docetaxel is shown in green. (G) Panel shows the eight drugs (see Fig. 1A) exhibiting a ≥10% increase in growth inhibition when combined with docetaxel at all three concentrations (0.1, 1 and 10 µM) in CP2 cells. Cytotoxicity was quantified by nuclear count using High Content Imaging Analysis (Operetta, Perkin Elmer). The percentage growth inhibition for each drug in combination with docetaxel relative to the DMSO control was calculated. n = 3 (technical replicates), mean values ± SD are shown, analysed by 2-way ANOVA with Sidak's multiple comparisons test, *p < 0.01, **p < 0.001, ***p < 0.0001. (H) Single drug (see Fig. 1A) exhibiting a ≥10% increase in growth inhibition when combined with

docetaxel at all three concentrations in SP1 cells. Analysis was carried out as for G. (H) Volcano plots showing the log2 fold change in cell number plotted against log10 p value for each drug concentration in both cell lines as indicated. Drugs with a fold change above 2 (log2 > 1) are shown in yellow/red scale; drugs with a fold change below 2 (log2 < 1) are shown in green. (J) SP1 cells were treated with mebendazole in combination with docetaxel and analysed as for G.

Figure S2. Data from validation screen on 4 of the 5 selected drugs. (A) CP2, SP1 and LNCaP cells were treated with the indicated drugs or DMSO control, in combination with docetaxel at a range of concentrations for 48 h. Cells were fixed, stained with DAPI, and cytotoxicity quantified by nuclear count using High Content Imaging Analysis (Operetta, Perkin Elmer). The percentage growth inhibition relative to the DMSO control for each docetaxel concentration (left panels), and the percentage cell survival after treatment with the target drugs alone (right panels) were calculated. n =3 (technical replicates), mean values ± SD are shown. (B) SP1 cells were treated with different drug combinations and the IncuCyte (Essen Bioscience) used to calculate the decrease in percentage cell survival drugs. n =3 (independent experiments), mean values ± SD are shown, analysed by 2-way ANOVA with Sidak's multiple comparisons test, ***p < 0.0001. Panel on the right shows the effects of docetaxel alone at doses used for combination treatment.

Figure S3. (A) CP2 cells were treated with different drug combinations, stained with annexin V and propidium iodide, and analysed by flow cytometry. The percentage of cells stained with annexin V is shown. n = 3 (independent experiments), mean values ± SD are shown, analysed

by 2-way ANOVA with Sidak's multiple comparisons test, *p < 0.01. (B, C) Cells were treated with docetaxel along with mebendazole (RWPE cells, B) or colchicine (SP1 and PC3 cells, C) at the indicated concentrations for 48 hours. Percentage growth inhibition was calculated for each drug combination using the CellTiter Glo Assay (Promega) as per the manufacturer's instructions. The Combination Index (CI) was calculated using CompuSyn software, where <1 indicates synergism, =1 is an additive effect, and >1 indicates antagonism. The scales and colour codes for the percentage growth inhibition and CI are shown on the left.

Figure S4. Example images of mitotic outcome. Scale bar is 50 µm.

Figure S5. Antiproliferative activity of liposomes encapsulating docetaxel (DOC, black), liposomes encapsulating mebendazole (MBZ, white), liposomes encapsulating docetaxel and mebendazole (DOC+MBZ, dark grey) and transferrin-bearing liposomes entrapping docetaxel and mebendazole (Tf DOC+MBZ, light grey) in PC-3M-Luc cells (top panel) and LNCaP cells (bottom panel). MTT assay was carried out as per the manufacturer's instructions. n = 12 from three independent experiments, mean values ± S.E.M are shown, analysed by 2-way ANOVA with Tukey's multiple comparisons test, *p < 0.05, **p < 0.001, ***p < 0.0001.

Figure S6. Tumour growth studies in a PC3M-Luc-G5 murine model. PC3M-Luc-G5 cells were subcutaneously injected to both flanks of male immunodeficient BALB/c mice, before intravenous administration of liposomes entrapping docetaxel and/or mebendazole (20 mg docetaxel and 180 mg mebendazole, respectively, per kg of body weight per injection. (A) Bioluminescence imaging in a PC3M-Luc-G5 tumour model showing the tumoricidal activity

of untargeted liposomes entrapping docetaxel and mebendazole (DOC + MBZ), liposomes entrapping docetaxel only (DOC) or mebendazole only (MBZ), transferrin-targeting liposomes entrapping docetaxel and mebendazole (Tf DOC + MBZ), empty liposomes and untreated controls. The scale indicates surface radiance (photons/s/cm^2/steradian). (B) Variations of the animal body weight throughout the treatment are shown. Treatments as for A. n=5, mean values ± SEM are shown.

Figure S1.

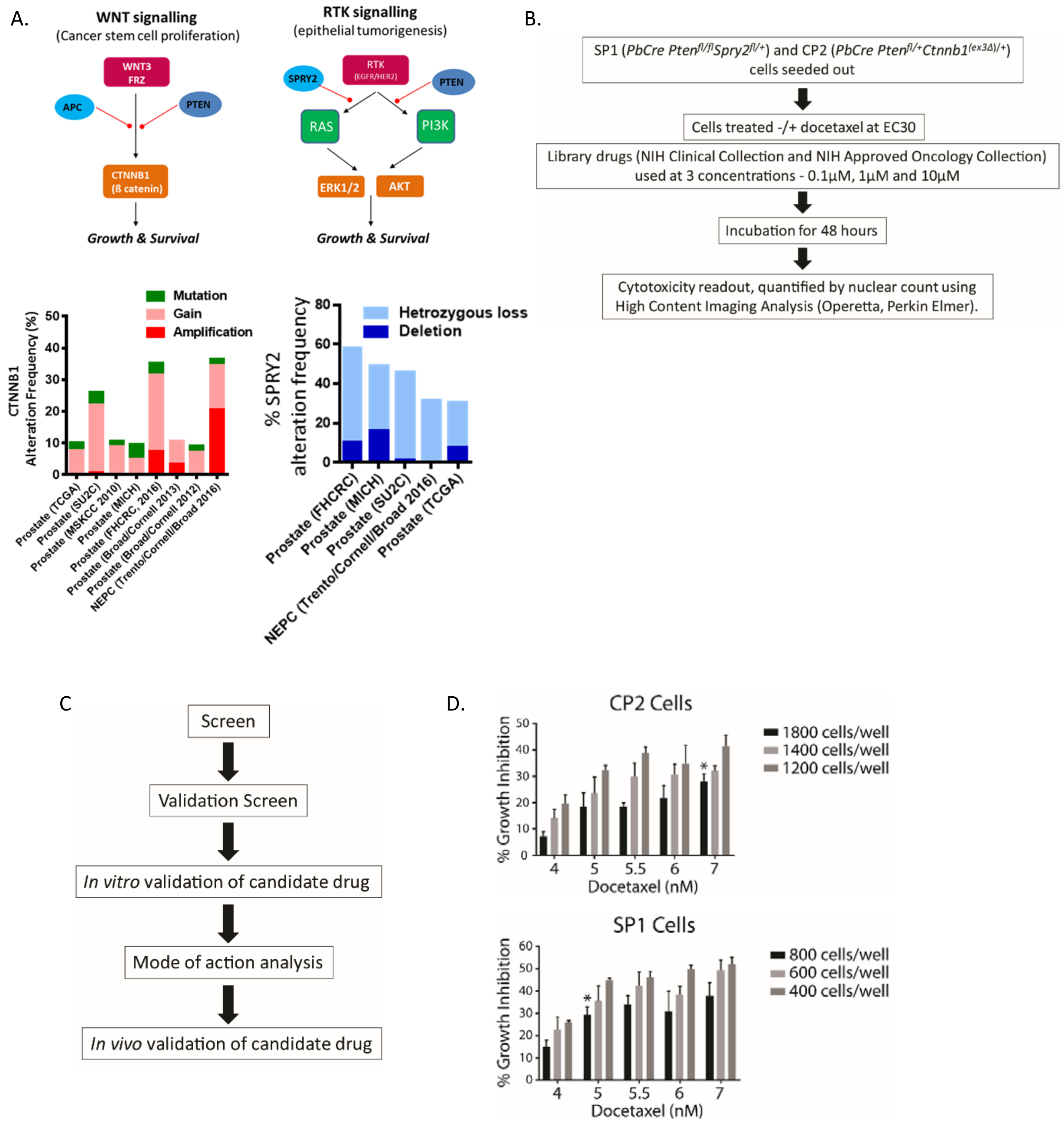
A.
WNT signalling
(Cancer stem cell proliferation)
WNT3
FRZ
APC
PTEN
CTNNB1
(ß catenin)
Growth & Survival
RTK signalling
(epithelial tumorigenesis)
RTK
(EGFR/HER2)
SPRY2
PTEN
RAS
PI3K
ERK1/2
AKT
Growth & Survival
Mutation
Gain
Amplification
CTNNB1
Alteration Frequency (%)
Prostate (TCGA)
Prostate (SU2C)
Prostate (MSKCC 2010)
Prostate (MICH)
Prostate (FHCRC, 2016)
Prostate (Broad/Cornell 2013)
Prostate (Broad/Cornell 2012)
NEPC (Trento/Cornell/Broad 2016)
Hetrozygous loss
Deletion
% SPRY2
alteration frequency
Prostate (FHCRC)
Prostate (MICH)
Prostate (SU2C)
NEPC (Trento/Cornell/Broad 2016)
Prostate (TCGA)
B.
SP1 (PbCre Pten fl/fl Spry2 fl/+) and CP2 (PbCre Pten fl/+ Ctnnb1 (ex3Δ)/+) cells seeded out
Cells treated -/+ docetaxel at EC30
Library drugs (NIH Clinical Collection and NIH Approved Oncology Collection) used at 3 concentrations - 0.1µM, 1µM and 10µM
Incubation for 48 hours
Cytotoxicity readout, quantified by nuclear count using High Content Imaging Analysis (Operetta, Perkin Elmer).
C
Screen
Validation Screen
In vitro validation of candidate drug
Mode of action analysis
In vivo validation of candidate drug
D.
CP2 Cells
% Growth Inhibition
Docetaxel (nM)
1800 cells/well
1400 cells/well
1200 cells/well
SP1 Cells
% Growth Inhibition
Docetaxel (nM)
800 cells/well
600 cells/well
400 cells/well

Figure S1 (Cont.)

E.

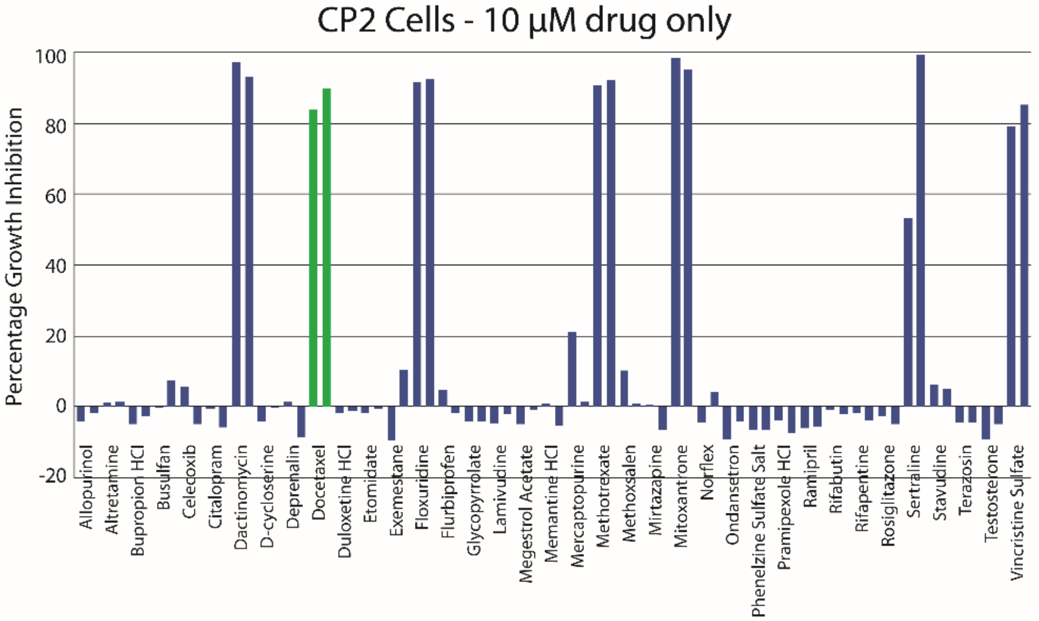

F.

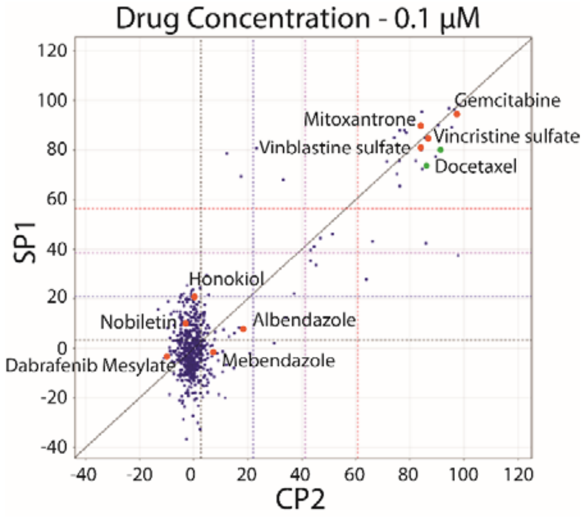

G.

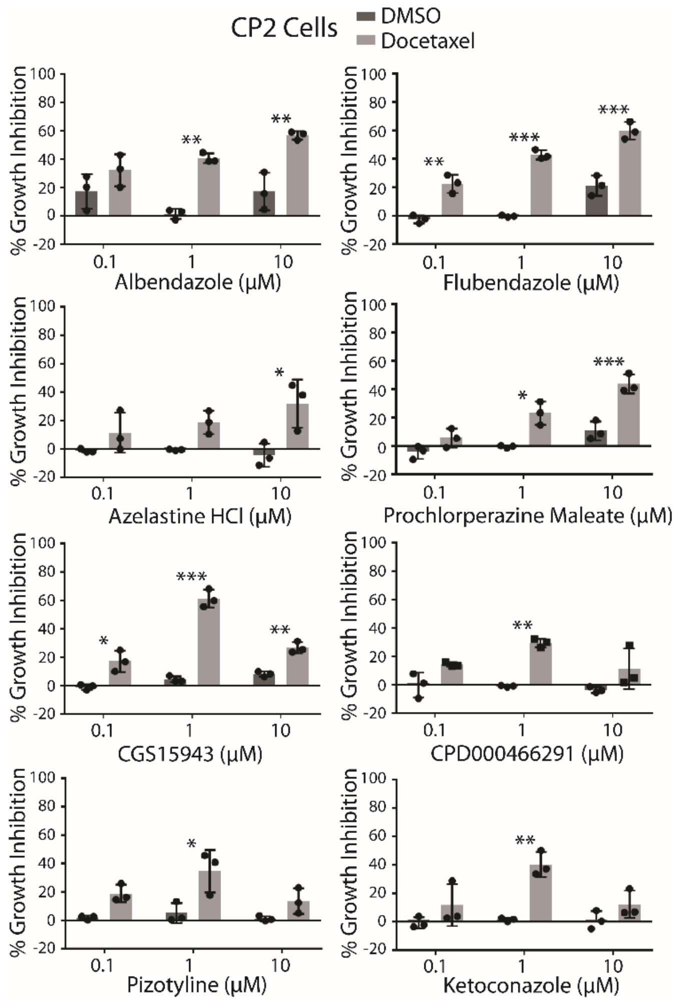

Figure S1. (Cont.)

H.

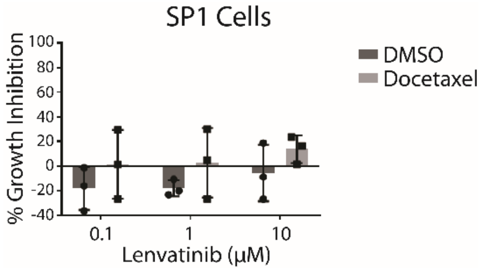

I.

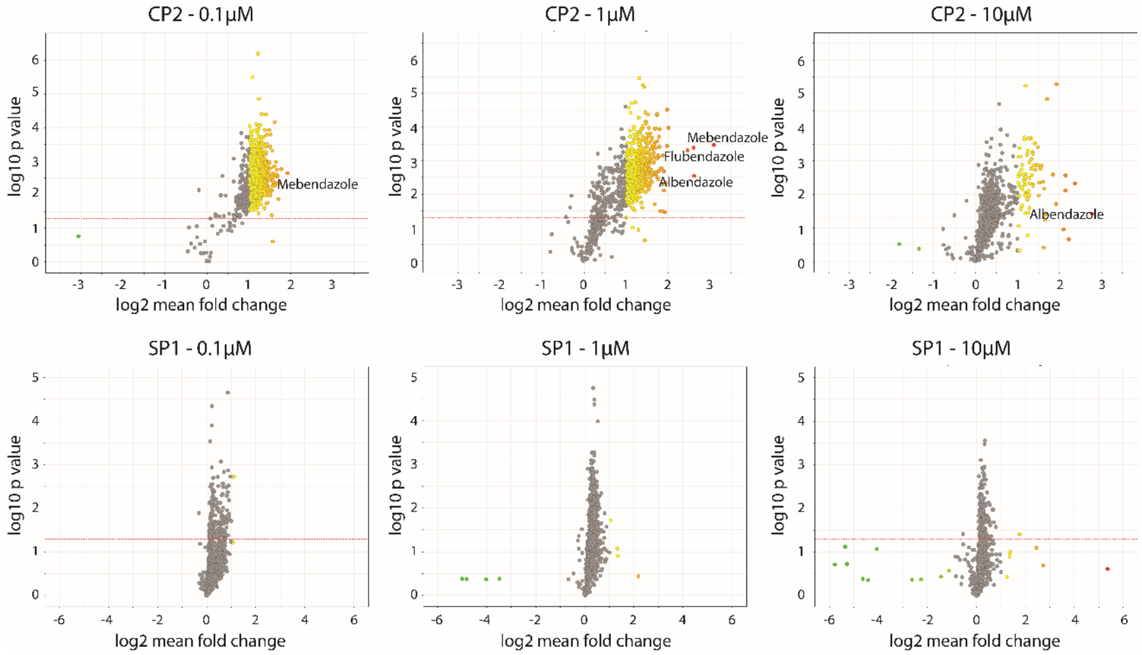

J.

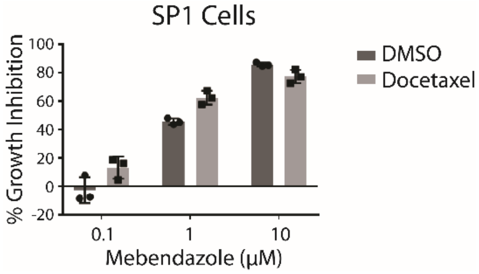

Figure S2.

A.

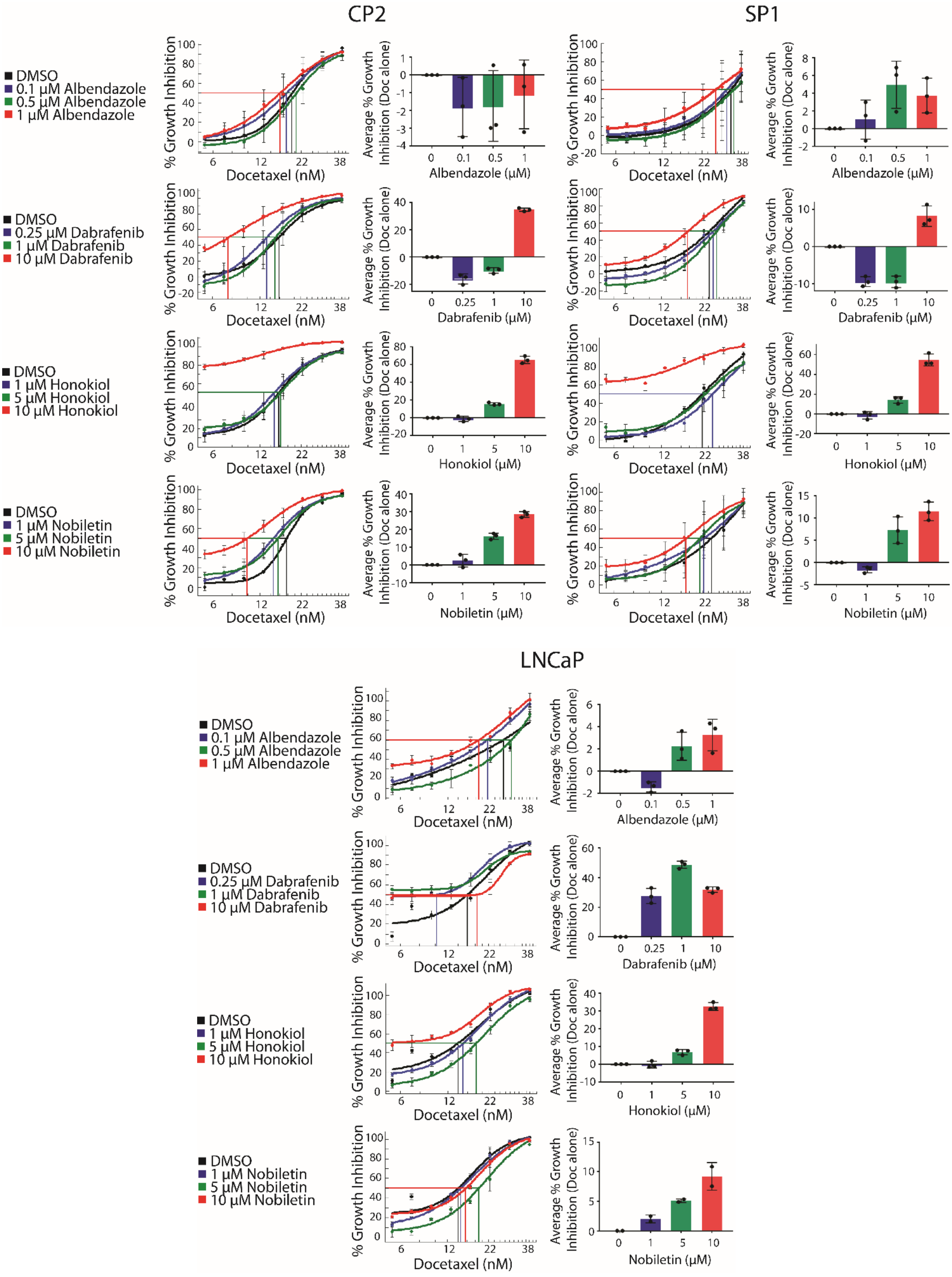
CP2
SP1
DMSO
0.1 µM Albendazole
0.5 µM Albendazole
1 µM Albendazole
0.25 µM Dabrafenib
1 µM Dabrafenib
10 µM Dabrafenib
1 µM Honokiol
5 µM Honokiol
10 µM Honokiol
1 µM Nobiletin
5 µM Nobiletin
10 µM Nobiletin
% Growth Inhibition
Docetaxel (nM)
Average % Growth Inhibition (Doc alone)
Albendazole (µM)
Dabrafenib (µM)
Honokiol (µM)
Nobiletin (µM)
LNCaP

Figure S2. (Cont.)

B.

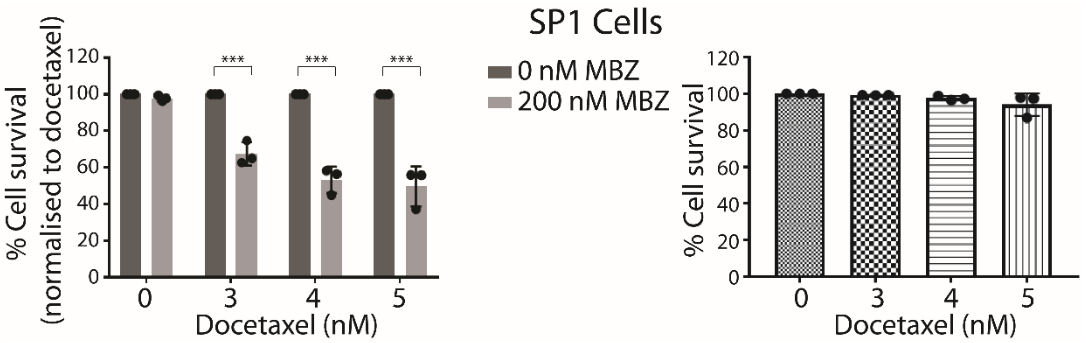

Figure S3.

A.

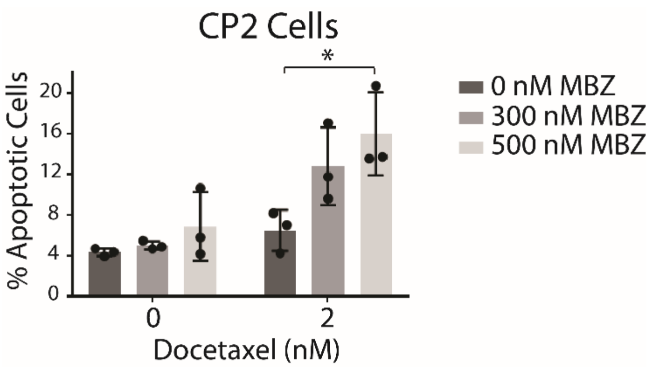

B.

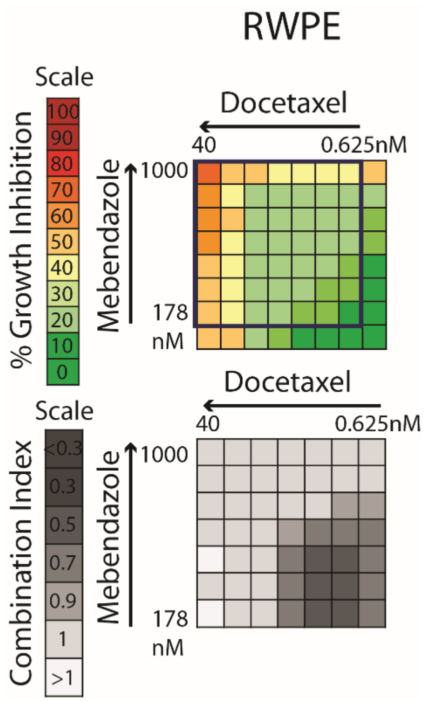

C.

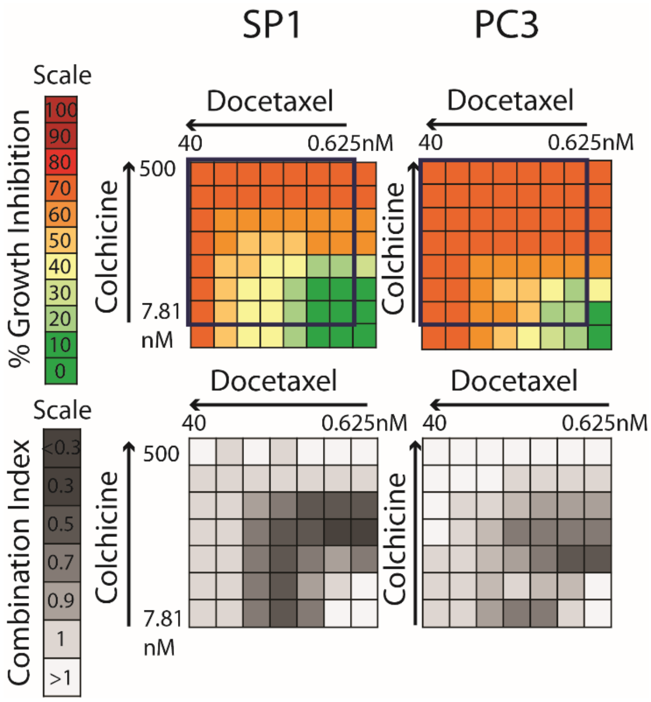

Figure S4.

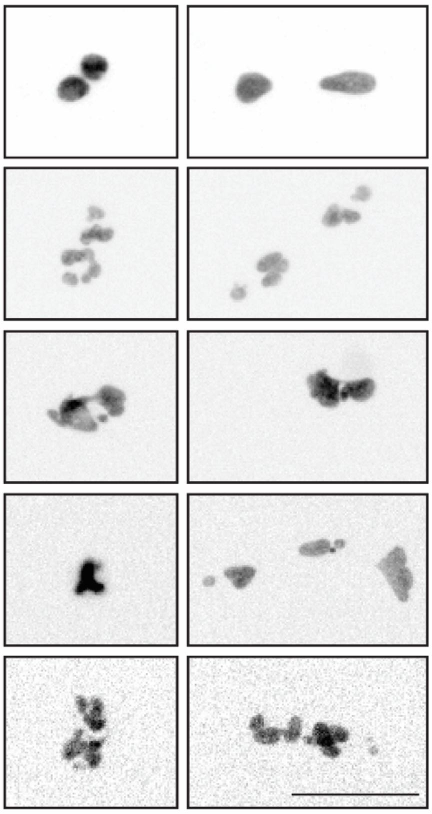

Successful mitosis = two intact nuclei moving apart

Two nuclei moving apart, but nuclei are fragmented or contain micronuclei

Two nuclei are formed but cytokinesis fails and both nuclei move together

multipolar division resulting in 3 or more daughter cells being formed

mitotic catastrophe leading to aneuploid cell with fragmented nucleus

Figure S5.

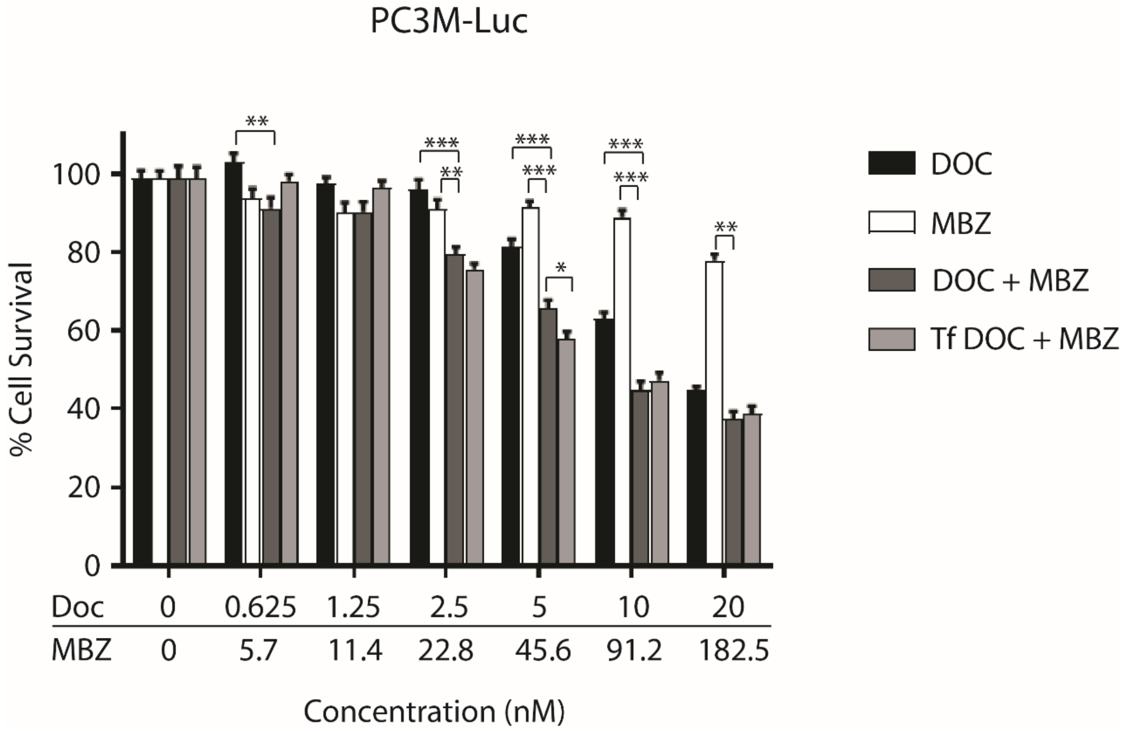

PC3M-Luc
% Cell Survival
100
80
60
40
20
0
**
***
**
***
***
*
***
***
**
DOC
MBZ
DOC + MBZ
Tf DOC + MBZ
Doc 0 0.625 1.25 2.5 5 10 20
MBZ 0 5.7 11.4 22.8 45.6 91.2 182.5
Concentration (nM)

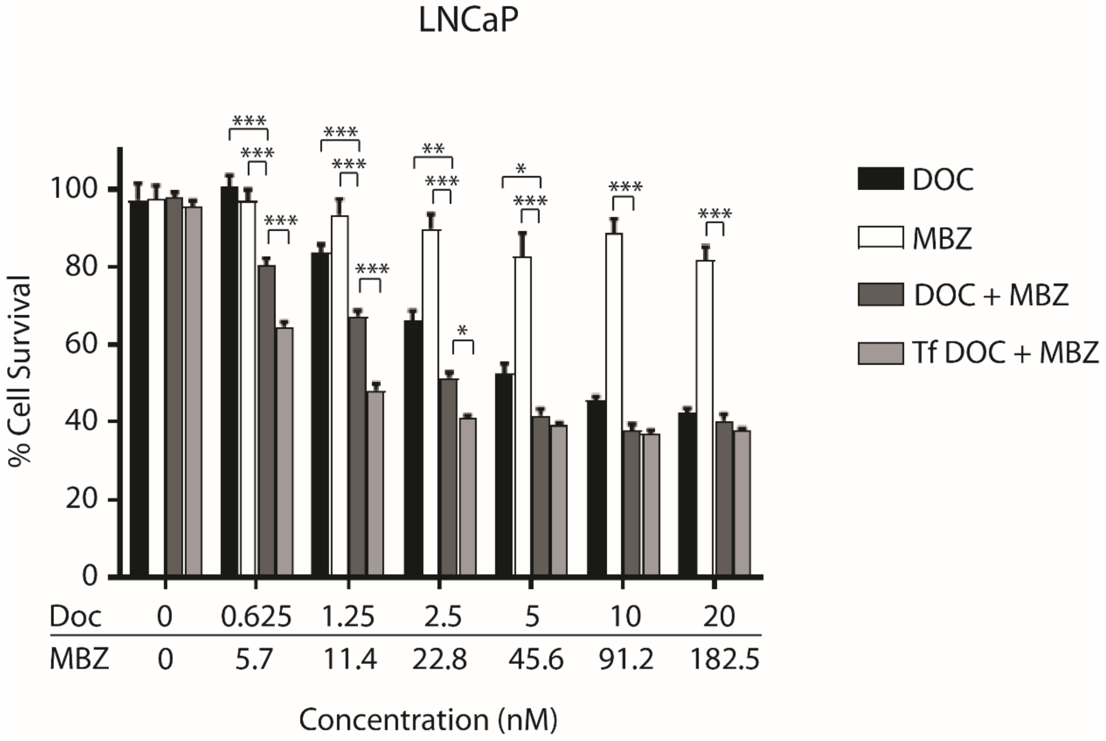

LNCaP
% Cell Survival
100
80
60
40
20
0
***
***
***
***
***
***
**
***
*
*
***
***
***
DOC
MBZ
DOC + MBZ
Tf DOC + MBZ
Doc 0 0.625 1.25 2.5 5 10 20
MBZ 0 5.7 11.4 22.8 45.6 91.2 182.5
Concentration (nM)

Figure S6.

A.

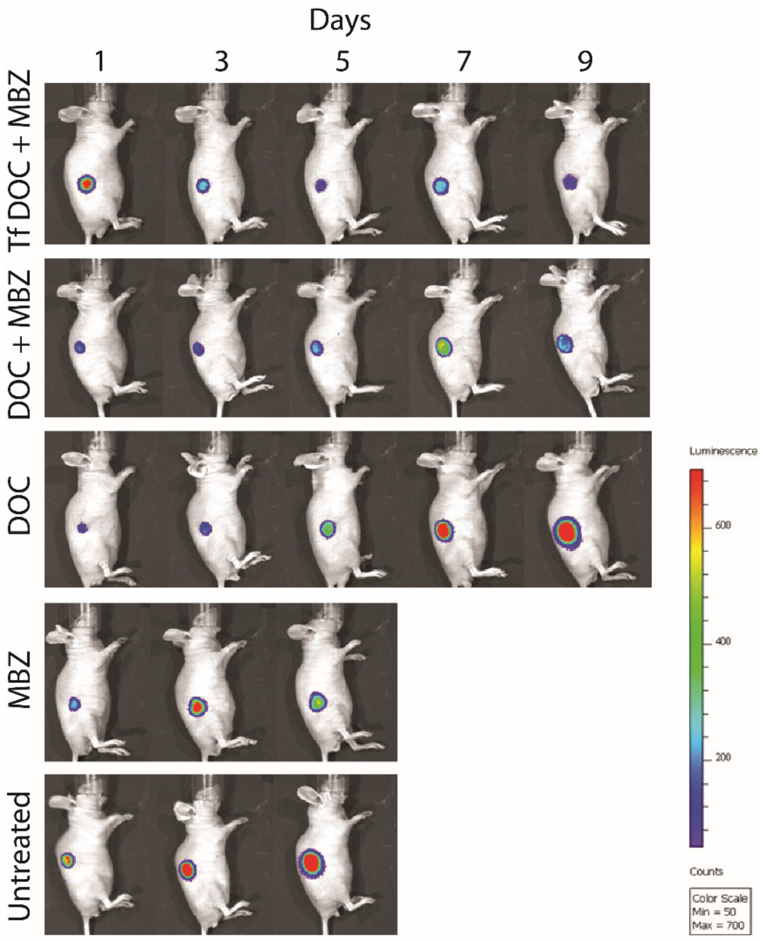

Days
1
3
5
7
9
Tf DOC + MBZ
DOC + MBZ
DOC
MBZ
Untreated
Luminescence
600
400
200
Counts
Color Scale
Min = 50
Max = 700

B.

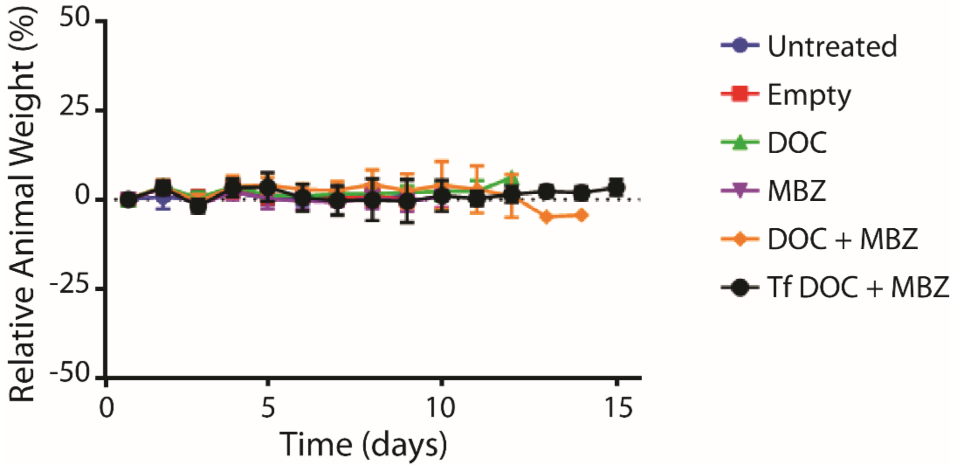

Relative Animal Weight (%)
50
25
0
-25
-50
0
5
10
15
Time (days)
Untreated
Empty
DOC
MBZ
DOC + MBZ
Tf DOC + MBZ

Table S1. Table of screen hits. Drugs from the NIH Clinical Collection are in upper case, drugs from the NIH Oncology Collection are in lower case. + indicates a >10% growth inhibition when combined with docetaxel compared to DMSO.

Drug	CP2			SP1		
	0.1 µM	1 µM	10 µM	0.1 µM	1 µM	10 µM
(-)-COTININE			+			
(+)-CIS-DILTIAZEM HCL		+	+			
(+/-)-EPINEPHRINE HCL	+	+				
(+/-)-NOREPINEPHRINE HCL						
11-DEOXYCORTISOL		+				
19-NORETHINDRONE	+					
19-NORETHINDRONE ACETATE	+		+			
1-BENZYLIMIDAZOLE		+				
2-(2-AMINOETHYL)PYRIDINE		+				
2,3-DIDEOXYCYTIDINE	+					
2,3-DIDEOXYINOSINE		+		+		
2-CHLORO-2-DEOXYADENOSINE						
2-CHLOROADENOSINE		+				
2-OXO-4-THIAZOLIDINE CARBOXYLIC ACID						
3,5,3-TRIIODOTHYRONINE	+					
3-DEOXYDENOSINE			+			
3-HYDROXY-N-METHYLMORPHINAN				+		
3-PYRIDINEMETHANOL						
5-AMINO-2-HYDROXY-BENZOIC ACID	+					
5-AZACYTIDINE	+		+			
5-FLUORO-2-PYRIMIDONE						
5-FLUOROCYTOSINE						
5-FLUOROURACIL						
5-METHOXYTRYPTAMINE						
5-NONYLOXYTRYPTAMINE						
6-AMINOINDAZOLE			+	+		
6-AZAURIDINE	+					
7-NITROINDAZOLE		+				
9-AMINO-1,2,3,4-TETRAHYDROACRIDINE HCL						
9-BETA-D-ARABINOFURANOSYLADENINE		+	+			
Abiraterone						+
ACARBOSE	+					
ACEBUTOLOL HCL	+					
ACETAZOLAMIDE						
ACETORPHAN				+		
ACITRETIN	+	+				
ACTARIT			+			
ACYCLOVIR						
Afatinib		+	+			
ALBALON	+					
ALBENDAZOLE	+	+	+		+	

Alectinib						+
ALFUZOSIN						
ALLEGRA						
Allopurinol	+		+	+		+
ALLOPURINOL						
ALOSETRON HCL		+				
ALPRAZOLAM	+				+	
ALTANSERIN	+	+		+		
ALTRETAMINE	+					
Altretamine						+
AM 404						
AM-251						
AMCINONIDE5						
Amifostine						
AMINOGLUTETHIMIDE	+			+		
AMINOLEVULINIC ACID	+					
Aminolevulinic acid hydrochloride			+	+		+
AMIODARONE HCL						
AMISULPRIDE				+		
AMLEXANOX						
AMLODIPINE BASE	+		+	+		
AMOXAPINE		+			+	
AMOXICILLIN CRYSTALLINE		+				
AMPICILLIN Na	+					
AMPIROXICAM			+			
ANAFRANIL						
ANAGRELIDE HCL						
ANASTROZOLE			+			
Anastrozole					+	
ANNOYLTIN	+					
ARGATROBAN				+		
ARIPIPRAZOLE		+				
Arsenic trioxide	+					
ARTANE	+		+			
ARTEMETHER		+				
ARTESUNATE						
ATENOLOL		+				
ATOMOXETINE HCL						
ATRACURIUM BESYLATE			+	+		
ATROPINE						
Axitinib	+		+			
Azacitidine				+	+	
AZASETRON		+				
AZATHIOPRINE			+			
AZELASTINE HCL	+	+	+			
AZITHROMYCIN		+				

BALSALAZIDE				+		
BECLOMETHASONE						+
BECLOMETHASONE DIPROPIONATE						
Belinostat				+		
BENACTYZINE HCL	+				+	
BENAZEPRIL HCL		+				
Bendamustine hydrochloride						
BENDROFLUAZIDE	+	+				
BENIDIPINE HCL						
BENPROPERINE PHOSPHATE		+		+		
BENTYL		+				
BENZBROMARONE			+			
BENZENEBUTANOIC ACID						
BESTATIN		+				
BETA-ESTRADIOL		+	+	+		
BETAMETHASONE						
BETAXOLOL HCL		+		+		
BICALUTAMIDE						
BIFEMELANE		+	+			
BIFONAZOLE		+				
BISOPROLOL FUMARATE						
Bleomycin sulfate						
Bortezomib						
Bosutinib		+		+	+	
BRIMONIDINE						
BRUCINE		+	+			
BUDESONIDE			+			
BUFLOMEDIL HCL						
BUMETANIDE		+	+			
BUPROPION HCL	+					
BUPROPION HCL	+					
BUSPAR						
BUSULFAN	+					
Busulfan						
Cabazitaxel						
Cabozantinib					+	
CALCIPOTRIOL		+		+		
CALCITRIOL		+			+	
CANTIL						
Capecitabine						
CAPSAICIN						
CAPTOPRIL						
CARBAMAZEPINE	+	+				
CARBIDOPA	+					
CARBINOXAMINE MALEATE						
Carboplatin						

CARDENE	+					
Carfilzomib						
CARISOPRODOL	+					
CARMOFUR						
Carmustine		+				+
CARVEDILOL					+	
CCPA			+			
CEFACLOR						
CEFATRIZINE PROPYLENE GLYCOL						
CEFAZOLIN Na SALT	+					
CEFDINIR						
CEFIXIME TRIHYDRATE						
CEFOTAXIME Na SALT	+					
CEFOXITIN Na SALT						
CEFPODOXIME PROXETIL						
CEFUROXIME		+				
Celecoxib			+		+	
CELECOXIB	+					
CEPHALEXIN MONOHYDRATE				+		
Ceritinib						
CERIVASTATIN NA	+					
CETIRIZINE						
CETRAXATE HCL						
CGS 12066B	+		+			
CGS 15943	+	+	+			
CHLORAMBUCIL	+		+	+		
Chlorambucil						
CHLORAMPHENICOL						
CHLORDIAZEPOXIDE		+				
CHLOROTHIAZIDE	+					
CHLOROXINE	+	+				
CHLORPHENIRAMINE	+					
CHLORPROPAMIDE	+	+				
CHLORTHALIDONE						
CHLORZOXAZONE	+			+		
CILASTATIN NA						
CIMETIDINE	+					
CINANSERIN		+				
CISAPRIDE	+	+				
Cisplatin						
CITALOPRAM		+				+
CITALOPRAM				+		
Cladribine						
CLARITHROMYCIN		+				
CLOBENPROPIT		+	+			
CLOBETASOL PROPIONATE					+	

Clofarabine				+		
CLOFAZIMINE		+	+	+		
CLONIDINE HYDROCL				+		
CLOPIDOGREL	+		+			
CLOTRIMAZOLE					+	
CLOZAPINE		+				
Cobimetinib				+	+	
COGENTIN MESYLATE						
CONJUGATED ESTROGENS			+			+
CORTELL					+	
CORTICOSTERONE						
CORTISONE	+		+			
CORTISONE ACETATE						
CPD000449323				+	+	
CPD000449325						
CPD000466279		+	+	+		
CPD000466286			+			
CPD000466291	+	+	+			
CPD000469631						
CPD000469633		+	+			
Crizotinib						
CROMOLYN Na	+					
Cyclophosphamide		+			+	+
CYPROHEPTADINE	+		+			
CYTARABINE		+		+		
Cytarabine hydrochloride						
CYTOXAN		+				
D-3-METHOXY-N-METHYLMORPHINAN HYDROBROMIDE				+		
Dabrafenib mesylate		+	+		+	
Dacarbazine			+			
Dactinomycin						
DACTINOMYCIN						
DANAZOL						
DANTROLENE Na SALT						
DAPSONE						
Dasatinib					+	
DAUNORUBICIN HCL				+		
Daunorubicin hydrochloride						
D-CYCLOSERINE				+		
D-CYCLOSERINE						
Decitabine						
DEFERIPRONE				+		
DEHYDROCHOLIC ACID			+			
DEHYDROEPIANDROSTERONE		+				
DEMECLOCYCLINE	+					
DEPO-MEDROL						

DEPRENALIN	+			+		
DEPRENALIN						
DESLORATADINE			+		+	
DESOXIMETASONE	+		+			+
DEXAMETHASONE						
DEXBROMPHENIRAMINE MALEATE		+				
DEXCHLORPHENIRAMINE MALEATE						
Dexrazoxane						
DIAZEPAM	+					
DIAZOXIDE	+	+				
DIBENZYLINE	+					
DICHLOROACETIC ACID	+	+		+		
DICLOFENAC Na						
DICLOXACILLIN Na		+		+		
DIFLUNISAL	+					
DIGOXIN	+					
DILANTIN						
DIPHENHYDRAMINE HCL						
DIPHENOXYLATE		+				
DIPHENYLCYCLOPROPENONE						
DIPYRIDAMOLE		+			+	
DL-PENICILLAMINE						
DMPP		+				
DOCETAXEL						
Docetaxel						
DOFETILIDE						
DOLASETRON MESYLATE		+	+			
DONEPEZIL		+			+	
DOXAPRAM HCL						
DOXAZOSIN		+				
DOXEPIN	+					
DOXEPIN HCL		+	+			
DOXORUBICIN HCL						+
Doxorubicin hydrochloride						
DOXYCYCLINE	+		+			
DOXYLAMINE SUCCINATE SALT						
DROPERIDOL		+		+		
DULOXETINE HCL		+	+	+		+
DULOXETINE HCL			+	+		
DUP 697						
DUREMESIN	+	+				
DUVADILAN						
EBSELEN						
ECONAZOLE NITRATE	+					
EDROPHONIUM CL						
EFAVIRENZ						

ENALAPRIL MALEATE	+					
ENALAPRILAT		+				
ENROFLOXACIN						
Enzalutamide			+		+	
EPIGALLOCATECHIN GALLATE						
EPIRUBICIN HCL						+
Epirubicin hydrochloride						
Erismodegib			+			
Erlotinib hydrochloride			+			+
ERYPED						
ESCITALOPRAM OXALATE		+				
ESMOLOL HCL	+	+				
ESOMEPRAZOLE MG		+				
ESTRADIOL VALERATE						
Estramustine phosphate sodium				+		+
ESZOPICLONE	+	+				
ETHACRYNIC ACID						
ETHAMBUTOL	+	+				
ETHIONAMIDE					+	
ETHYLESTRENOL			+	+		
ETHYNYLESTRADIOL						
ETODOLAC						
ETOMIDATE			+			
ETOMIDATE		+				
ETOMOXIR						
ETOPOSIDE						
Etoposide						
Everolimus						
EVISTA	+	+		+		
EXEMESTANE						
Exemestane			+		+	
EZETIMIBE						
FAMCICLOVIR		+				
FAMOTIDINE	+	+		+		
FELBAMATE				+		
FELODIPINE	+	+				
FENOFIBRATE						
FENOLDOPAM MESYLATE						
FENPIVERINIUM BROMIDE	+					
FINASTERIDE		+				
FLECAINIDE ACETATE	+	+			+	
Floxuridine						
FLOXURIDINE						
FLUBENDAZOLE	+	+	+		+	
FLUCONAZOLE	+					
FLUDARABINE						

Fludarabine phosphate					+	
FLUMADINE						
FLUMAZENIL	+	+				
FLUNISOLIDE						
FLUOCINOLONE ACETONIDE						
FLUOCINOLONE ACETONIDE 21-ACETATE						
FLUOROMETHOLONE					+	
Fluorouracil					+	
FLUPERLAPINE	+					
FLUPHENAZINE DIHYDROCHLORIDE			+			
FLURBIPROFEN			+	+		
FLURBIPROFEN						
FLUTAMIDE	+		+			
FLUTICASONE PROPIONATE						
FLUVASTATIN	+				+	
FLUVOXAMINE	+			+		
FOLIC ACID	+		+			
FORMOTEROL FUMARATE DIHYDRATE						
FTORAFUR		+				
Fulvestrant		+	+	+		
FUROSEMIDE		+				
GABEXATE MESILATE		+				
GALANTHAMINE		+		+	+	
GANCICLOVIR	+					
GATIFLOXACIN						
Gefitinib			+		+	+
Gemcitabine hydrochloride						
GEMFIBROZIL						
GLIMEPIRIDE				+		
GLIPIZIDE						
GLYBURIDE			+			
GLYCOPYRROLATE				+		
GLYCOPYRROLATE						
GOSERELIN ACETATE		+				
GR 89696		+				
GRANISETRON HCL						
GRISEOFULVIN			+			+
GUANIDINE						
HALOMETASONE MONOHYDRATE						+
HALOPERIDOL						
HEXACHLOROPHENE						
HMBA		+		+		
HOMOHARRINGTONINE						
HOMOVERATRYLAMINE						
HONOKIOL		+	+			
HTMT						

HUPERZINE A		+				
HYDROCHLOROTHIAZIDE						
HYDROCORTISONE						
HYDROCORTISONE HEMISUCCINATE						
HYDROFLUMETHIAZIDE	+					
Hydroxyurea						+
HYPEROSIDE				+		
Ibrutinib			+			
IBUPROFEN	+					
ICARIIN						
IDARUBICIN HCL						
Idarubicin hydrochloride						
IDEBENONE			+			
Idelalisib			+			+
IFENPRODIL						
Ifosfamide				+		
Imatinib						
IMATINIB MESYLATE	+		+	+		
Imiquimod						+
IMODIUM			+			
INDAPAMIDE						
INDATRALINE		+	+	+		
INDERAL		+				
INDINAVIR SULPHATE						
INDIRUBIN						
INDOMETHACIN	+					
INTROPIN						
IPIDACRINE				+		
IPRATROPIUM BROMIDE MONOHYDRATE						
IPRIFLAVONE		+			+	
IRBESARTAN				+		
IRINOTECAN HCL TRIHYDRATE		+				
Irinotecan hydrochloride					+	
IRSOGLADINE MALEATE			+	+		
ISONIAZID						
ISOQUERCITRIN		+				
ISOTRETINOIN						
ISRADIPINE		+	+	+		
ISUPREL	+					
ITAVASTATIN CA						
ITOPRIDE HCL			+			
ITRACONAZOLE					+	
Ixabepilone						
Ixazomib citrate				+		
KEMADRIN						
KETOCONAZOLE	+	+	+	+		

KETOPROFEN	+				+	
KETOROLAC TROMETHAMINE				+		
KETOTIFEN FUMARATE			+			
KITASAMYCIN		+	+	+		
L-694,247						
LABETALOL HCL						
LACIDIPINE						
LAMIVUDINE	+		+			
LAMIVUDINE		+				
LAMOTRIGINE	+			+		
LANSOPRAZOLE		+		+		
Lapatinib			+	+		
LATANOPROST	+	+				
Lenalidomide				+		
Lenvatinib			+	+	+	+
LETROZOLE		+		+		
Letrozole						
LEVETIRACETAM				+		
LEVOCETIRIZINE						
LEVOFLOXACIN						
LEVONORGESTREL		+				
LEVOSULPIRIDE		+				
LIDOCAINE				+		
LINCOMYCIN HCL		+				
LINEZOLID				+		
L-NMMA						
LOBELINE HCL	+					
LOFEPRAMINE						
LOFEXIDINE HCL						
LOMERIZINE DIHCL				+		
LOMIFYLLINE						
Lomustine				+		
LORATADINE		+	+			
LORAZEPAM			+			
LOSARTAN POTASSIUM						
LOTEPREDNOL ETABONATE				+		
LOVASTATIN				+		
LOXAPINE SUCCINATE		+	+	+		
LOXOPROFEN Na		+				
L-THYROXINE	+					
MAFENIDE ACETATE	+					
MALTOL						
MAPROTILLINE HCL			+			+
MAXOLON	+					
MEBENDAZOLE	+	+		+	+	
Mechlorethamine hydrochloride						

MECILLINAM						
MECLOMEN	+					
MEDROXYPROGESTERONE		+		+	+	
MEDROXYPROGESTERONE 17-ACETATE						+
MEDRYSONE	+	+			+	+
MEFENAMIC ACID						
MEFLOQUINE HCL						
Megestrol acetate		+	+	+		
MEGESTROL ACETATE		+				
MELOXICAM	+	+				
Melphalan hydrochloride						
MEMANTINE HCL		+				
MEMANTINE HCL						
MEPIRIZOLE	+	+				
MEPIVACAINE HCL						
Mercaptopurine						
MERCAPTOPURINE						
MEROPENEM		+		+		
MESNA	+					
MESORIDAZINE						
MESTANOLONE						
MESTINON	+			+		
MESTRANOL		+				
METAPROTERENOL	+					
METHAZOLAMIDE						
METHIMAZOLE						
METHOCARBAMOL	+					
METHOTREXATE						
Methotrexate						
Methoxsalen						+
METHOXSALEN						
METHYLANDROSTENEDIOL						
METHYLDOPA	+					
METHYLPREDNISOLONE						
METHYLTESTOSTERONE	+					
METRONIDAZOLE	+					
METYLPERON				+		
METYRAPONE			+			
MEVASTATIN						
MEXITIL		+				
MICONAZOLE NITRATE						
MICROPENIN	+			+		
MIDAZOLAM HCL		+				
MIFEPRISTONE		+	+		+	
MIGLITOL		+				
MILNACIPRAN	+					

MILRINONE						
MINOCYCLINE HCL	+					
MINOXIDIL		+				
MIOCHOL						
MIRTAZAPINE		+				
MIRTAZAPINE						
Mitomycin				+		
Mitotane						
Mitoxantrone						
MITOXANTRONE						
MK 886						
MOBAN						
MOCLOBEMIDE		+	+			
MODAFINIL	+					
MONTELUKAST NA						
MOSAPRIDE CITRATE		+			+	
MOXIFLOXACIN HCL		+				
MOXONIDINE HCL				+		
MUPIROCIN						
NABUMETONE	+					
NADOLOL						
NAFADOTRIDE	+		+			+
NAFCILLIN Na	+					
NAFTOPIDIL		+				
NALBUPHINE	+	+				
NALIDIXIC ACID					+	
NALOXONE HCL						
NALTREXONE HCL						
NALTRINDOLE				+		+
NANDROLONE						
NAPROXEN Na			+			
NATEGLINIDE						
NEFAZODONE						
Nelarabine						
NELFINAVIR MESYLATE						
N-ETHYL-O-CROTONOTOLUIDIDE	+					
NIALAMIDE						
NICORANDIL						
NICOTINAMIDE						
NICOTINE						
NICOTINIC ACID	+					
NIFEDIPINE			+		+	
NIFEKALANT HCL						
Nilotinib		+	+			
NIMETAZEPAM		+				
NIMODIPINE			+			

NISOLDIPINE		+				
NITAZOXANIDE						
NITRAZEPAM						
NITRENDIPINE	+	+			+	
NITROFURANTOIN	+					
NIZATIDINE				+		
NOBILETIN		+	+		+	
NORFLEX						
NORFLEX						
NORFLOXACIN		+				
NORNICOTINE		+				
NORPACE		+				
NORPRAMIN		+				
NOVOCAIN	+					
OFLOXACIN		+				
OLANZAPINE			+	+		
Olaparib					+	
OLIGOMYCIN C		+	+		+	
OLMESARTAN MEDOXOMIL				+		
OLOPATADINE HCL						
Omacetaxine mepesuccinate						
OMEPRAZOLE		+				
ONDANSETRON						
ONDANSETRON						
ORLISTAT		+				
ORMETOPRIM						
ORNIDAZOLE		+				
Osimertinib					+	
OTENZEPAD						
Oxaliplatin				+		
OXAPROZIN		+				
OXICONAZOLE NITRATE		+		+		
OXYBUTYNIN CL	+					
OXYMETHOLONE	+					
OXYPHENONIUM BROMIDE						
OXYTETRACYCLINE HCL						
OZAGREL MONOHYDROCHLORIDE						
Paclitaxel						
Palbociclib				+		+
PALONOSETRON HCL			+			
PAMELOR	+					
PANCURONIUM		+				
Panobinostat						
PANTOPRAZOLE Na SALT						
PARECOXIB NA				+		
PAROXETINE		+	+			

PAROXETINE			+			
PAROXETINE		+				
Pazopanib hydrochloride			+		+	
PAZUFLOXACIN			+			
PD 81723		+		+		
PEFLOXACIN MESYLATE						
Pemetrexed, Disodium salt, Heptahydrate						
PEMOLINE		+				
PENCICLOVIR		+				
PENICILLIN V						
Pentostatin					+	
PENTOXIFYLLINE					+	
PERGOLIDE MESYLATE		+	+			
PEROSPIRONE HCL	+		+			
PERPHENAZINE			+			+
PFIZERPEN		+				
PHENELZINE SULFATE SALT						
PHENELZINE SULFATE SALT						
PHENERGAN	+		+			
PHENOTHIAZINE						
PHENPROBAMATE						
PHENTOLAMINE HCL						
PHYLLOQUINONE	+					
PHYSOSTIGMINE	+					
PICEID						
PICROTOXININ	+					
PIDOTIMOD	+		+	+		
PILOCARPINE HCL						
PINACIDIL MONOHYDRATE		+				
PINDOLOL						
PIOGLITAZONE HCL						
PIPERACILLIN Na SALT						
Pipobroman				+		
PIRENPERONE		+	+		+	
PIRIBEDIL		+				
PIROXICAM						
PIZOTYLINE	+	+	+			
Plerixafor			+			
Plicamycin					+	
PODOFILOX						
Pomalidomide				+		
Ponatinib				+		
Pralatrexate						
PRAMIPEXOLE HCL			+	+		
PRAMIPEXOLE HCL						
PRAVASTATIN Na				+		

PRAZIQUANTEL						
PRAZOSIN			+			
PREDNISOLONE						
PREDNISOLONE ACETATE						
PREDNISOLONE Na SUCCINATE				+		
PREDNISONE						
PRILOCAINE HCL						
PRIMAQUINE DIPHOSPHATE	+		+			
PRIMIDONE						
PRISCOLINE	+					
PRO-AMATINE						
PRO-BANTHINE	+			+		
PROBENECID						
PROCARBAZINE HCL		+				
Procarbazine hydrochloride						+
PROCHLORPERAZINE MALEATE	+	+	+			
PROGESTERONE						
PRONESTYL	+	+				
PROPOFOL		+				
PROPYLTHIOURACIL						
PROSTAGLANDIN E1			+			
PROXYMETACAINE		+				
PROZAC						
PTEROSTILBENE		+	+			
PYRAZINAMIDE						
PYRIDINE-2-ALDOXIME METHOCL	+		+			+
PYRIMETHAMINE	+			+		
QUETIAPINE HEMIFUMARATE						
QUINAPRIL HCL						
QUINIDINE HCL MONOHYDRATE						
R(+)-SCH-23390 HCL						
RABEPRAZOLE	+		+			
RACLOPRIDE		+				
Raloxifene					+	
RALTITREXED						
RAMIPRIL	+		+	+		
RAMIPRIL						
RANITIDINE HCL	+		+			
RANOLAZINE DIHCL		+				
Regorafenib					+	
REMACEMIDE MONOHYDROCL	+	+				
REPAGLINIDE				+		
RESVERATROL			+			
RETINOIC ACID	+					
RIBAVIRIN		+				
RIFABUTIN		+	+		+	+

RIFABUTIN		+			+	
RIFAMPICIN	+					
RIFAPENTINE		+	+			
RIFAPENTINE			+			
RIFAXIMIN			+	+		+
RILUZOLE						
RIMCAZOLE		+	+			
RISPERIDONE		+				
RITONAVIR			+	+		
RIZATRIPTAN BENZOATE		+				
ROFECOXIB				+		
ROLIPRAM		+	+			+
ROLITETRACYCLINE			+			+
Romidepsin						
ROPIVACAINE HCL						
ROSIGLITAZONE HCL		+				
ROSIGLITAZONE HCL						
ROXATIDINE ACETATE HCL						
RU 24969		+				
RUFLOXACIN HCL		+				
RUTIN		+		+		
RYTHMOL						
S(-)-TIMOLOL MALEATE	+					
SALBUTAMOL SULFATE	+					
SALMETEROL			+			
SAQUINAVIR MESYLATE		+				
SB 205607						
SCOPOLAMINE HYDROBROMIDE						
SDM25N		+				
SECNIDAZOLE	+					
SECOISOLARICIRESINOL						
SERTRALINE			+		+	+
SERTRALINE		+				
SIBUTRAMINE						
SIBUTRAMINE HCL		+				
SIMVASTATIN	+	+		+		
Sirolimus						
SKF 83566	+			+		
SONAZINE						
Sorafenib			+			
SOTALOL HCL		+				
SPECTINOMYCIN		+				
SPECTINOMYCIN DIHCL PENTAHYDRATE		+			+	
SPIRONOLACTONE			+			
SR 57,227A	+					
STANOZOLOL			+			

STAVUDINE		+	+			
STAVUDINE		+	+			
STIRIPENTOL						
Streptozocin				+		+
SULFACETAMIDE					+	
SULFAMETHOXAZOLE	+	+				
SULFASALAZINE		+				
SULFINPYRAZONE						
SULFISOXAZOLE	+					
SULINDAC				+		
SUMATRIPTAN SUCCINATE						
Sunitinib			+			
SYMMETREL						
SYNEPHRINE						
TACROLIMUS		+			+	
TADALAFIL						
TAMOXIFEN						
Tamoxifen citrate				+	+	
TAXIFOLIN-(+)		+				
TAXIFOLIN-(+/-)			+			
TEGASEROD MALEATE		+				
TELITHROMYCIN		+	+		+	+
TELMISARTAN		+	+		+	
TEMOZOLOMIDE				+		
Temozolomide						
Temsirolimus						+
Teniposide						
TERAZOSIN		+		+		
TERAZOSIN	+					
TERBINAFINE HCL		+				
TERBUTALINE SULFATE		+				
TESTOSTERONE						
TESTOSTERONE						
TETRACYCLINE						
TETRAETHYLTHIURAM DISULFIDE	+		+			
TFMPP						
THALIDOMIDE	+					
Thalidomide		+		+		
THEOPHYLLINE						
THIABENDAZOLE	+					
Thioguanine						
THIORIDAZINE HCL						
Thiotepa	+			+		
THIOTHIXENE	+		+			
TIAGABINE HCL						
TIBOLONE						

TICLOPIDINE HCL		+				
TINIDAZOLE						
TIZANIDINE HCL						
TOCAINIDE				+		
TOFRANIL						
TOLAZAMIDE		+				
TOLBUTAMIDE						
TOLTERODINE TARTRATE	+	+				
TOMELUKASTUM						
TOPIRAMATE						
TOPOTECAN HCL						
Topotecan hydrochloride				+		
TORASEMIDE					+	
TOREMIFENE CITRATE		+				
TOSUFLOXACIN TOSYLATE						
TRAMADOL		+	+			
Trametinib						
TRANILAST						
TRAZODONE HCL					+	
TREMULACIN		+	+			
Tretinoin						+
TRIAMCINOLONE ACETONIDE	+					
TRIAMTERENE		+				
TRICLABENDAZOLE		+				
TRICLOSAN						
Triethylenemelamine				+		
Trifluridine						
TRILEPTAL			+			
TRIMEBUTINE MALEATE		+			+	
TRIMETHOPRIM	+					
TRIPELENNAMINE HCL		+				
TRIPFLUOPERAZINE HCL						
TRIPTOLIDE						
TROPICAMIDE						
TROPISETRON HCL		+				
TROXIPIDE		+	+	+		
TRYPTOLINE			+	+		
TYZINE						
Uracil mustard						
URAPIDIL HCL						
URECHOLINE		+				
Uridine triacetate						
URSODEOXYCHOLIC ACID						
VALACICLOVIR HCL		+				
VALDECOXIB						
VALPROIC ACID		+				

Valrubicin						
VALSARTAN		+				
Vandetanib					+	
VARDENAFIL CITRATE			+		+	
VECURONIUM BROMIDE						+
Vemurafenib	+		+		+	
VENLAFAXINE HCL						
VESAMICOL HCL				+		
Vinblastine sulfate						
VINCRISTINE SULFATE						
Vincristine sulfate						
VINDESINE SULFATE						
VINORELBINE BITATRATE				+		
Vinorelbine tartrate						
VIRAMUNE						
Vismodegib			+			
VISTARIL PAMOATE						+
VORICONAZOLE						
Vorinostat						
WARFARIN Na						
WESTCORT					+	
XANTHINOL NICOTINATE				+		
ZACOPRIDE		+				
ZAFIRLUKAST		+	+	+		
ZALEPLON						
ZARDAVERINE			+			
ZERANOL		+	+			
ZIDOVUDINE		+	+			
ZILEUTON						
Zoledronic acid					+	
ZOLMITRIPTAN		+				
ZOLPIDEM TARTRATE						
ZONISAMIDE						

Table S2. Current uses and cancer associations for drugs selected for validation.

	Current Use	Use in Cancer Studies	Prostate Cancer Associations
Albendazole	Anti-parastic drug; inhibits microtubule assembly	Inhibits proliferation of HNSCC [1], ovarian [2], gastric cancer [3] cell lines; improves survival of HCT-116 tumour bearing mice in combination with 2-Methoxyestradiol [4]	Synergistic with colchicine and 2-Methoxyestradiol in DU145 [4]
Dabrafenib Mesylate	B-Raf V600E inhibitor	Used to treat metastatic melanoma; approved in 2017 for treatment of NSCLC in combination with Trametanib [5]; currently being tested for thyroid cancer treatment [6]	No previous indications
Honokiol	Isolated from bark; has anti-inflammatory, anti-carcinogenic, anti-angiogenic properties	Inhibits growth of multiple types of cancer cells both in vitro and in vivo [7]	Xenograft models treated in combination with docetaxel have lower PSA levels [8]; inhibits bone metastatic growth of PC cells [9]; induces ROS-mediated autophagy in PC cells [8]
Mebendazole	Anti-parasitic drug, inhibits microtubule assembly	Shown to inhibit proliferation of many cancer cell types and/or have efficacy *in vivo* in many cancer types including melanoma [10], HNSCC [11] and colon [12]; Clinical trials for glioblastoma – no results to date	No previous indications; other family members have tested in vitro and shown to inhibit PC cell growth [13]
Nobiletin	Flavonoid isolated from citrus peel; improves memory impairment [14, 15]	Inhibits proliferation and induces apoptosis of ovarian cancer cells [16]	Suppresses cell viability through AKT pathways in PC3 and DU145 [17]

Table S3.

	SP1	CP2	LNCaP	PC3	CWR22	RWPE
EC30 Docetaxel (nM)	3.5	1	1	2	7.5	10
EC30 Mebendazole (nM)	300	300	280	320	520	600

Table S4. Physicochemical characterization of liposomes (n = 9 from three independent experiments). (Data was represented as Mean ± S.E.M). (TF, transferrin; LIPO, liposome; MBZ, mebendazole; DTX, docetaxel)

**Formulation**	**Size (nm)**	**Polydispersity index (PDI)**	**Zeta potential (mV)**	**Encapsulation efficiency (%)**
LIPO MBZ	118.4 ± 0.47	0.37 ± 0.004	-13.9 ± 0.45	44
LIPO DTX	73.4 ± 0.17	0.16 ± 0.003	-21.4 ± 0.45	80.4
LIPO DTX + MBZ	133.2 ± 0.71	0.39 ± 0.003	-16.0 ± 0.86	MBZ: 43<br>DTX: 83.4
TF LIPO DTX + MBZ	137.8 ± 0.53	0.45 ± 0.01	-36.01111 ± 0.31554	MBZ: 44.3<br>DTZ: 83.7

Table S5. Median progression free survival of mice treated with untargeted liposomes entrapping docetaxel and mebendazole (DOC + MBZ), liposomes entrapping docetaxel only (DOC) or mebendazole only (MBZ), transferrin-targeting liposomes entrapping docetaxel and mebendazole (Tf DOC + MBZ), empty liposomes and untreated controls.

Treatment	Median Progression Free Survival (days)
Untreated	1.5
Empty	2
DOC	5
MBZ	3.5
DOC + MBZ	10
Tf DOC + MBZ	10.5

References

1. Ghasemi F, Black M, Vizeacoumar F, Pinto N, Ruicci KM, Le C*, et al.* Repurposing Albendazole: new potential as a chemotherapeutic agent with preferential activity against HPV-negative head and neck squamous cell cancer. *Oncotarget* 2017, **8**(42)**:** 71512-71519.

2. Chu SW, Badar S, Morris DL, Pourgholami MH. Potent inhibition of tubulin polymerisation and proliferation of paclitaxel-resistant 1A9PTX22 human ovarian cancer cells by albendazole. *Anticancer Res* 2009, **29**(10)**:** 3791-3796.

3. Zhang X, Zhao J, Gao X, Pei D, Gao C. Anthelmintic drug albendazole arrests human gastric cancer cells at the mitotic phase and induces apoptosis. *Exp Ther Med* 2017, **13**(2)**:** 595-603.

4. Ehteda A, Galettis P, Pillai K, Morris DL. Combination of albendazole and 2-methoxyestradiol significantly improves the survival of HCT-116 tumor-bearing nude mice. *BMC Cancer* 2013, **13:** 86.

5. Odogwu L, Mathieu L, Blumenthal G, Larkins E, Goldberg KB, Griffin N*, et al.* FDA Approval Summary: Dabrafenib and Trametinib for the Treatment of Metastatic Non-Small Cell Lung Cancers Harboring BRAF V600E Mutations. *Oncologist* 2018, **23**(6)**:** 740-745.

6. Subbiah V, Kreitman RJ, Wainberg ZA, Cho JY, Schellens JHM, Soria JC*, et al.* Dabrafenib and Trametinib Treatment in Patients With Locally Advanced or Metastatic BRAF V600-Mutant Anaplastic Thyroid Cancer. *J Clin Oncol* 2018, **36**(1)**:** 7-13.

7. Prasad R, Katiyar SK. Honokiol, an Active Compound of Magnolia Plant, Inhibits Growth, and Progression of Cancers of Different Organs. *Adv Exp Med Biol* 2016, **928:** 245-265.

8. Hahm ER, Karlsson AI, Bonner MY, Arbiser JL, Singh SV. Honokiol inhibits androgen receptor activity in prostate cancer cells. *Prostate* 2014, **74**(4)**:** 408-420.

9. Shigemura K, Arbiser JL, Sun SY, Zayzafoon M, Johnstone PA, Fujisawa M*, et al.* Honokiol, a natural plant product, inhibits the bone metastatic growth of human prostate cancer cells. *Cancer* 2007, **109**(7)**:** 1279-1289.

10. Simbulan-Rosenthal CM, Dakshanamurthy S, Gaur A, Chen YS, Fang HB, Abdussamad M*, et al.* The repurposed anthelmintic mebendazole in combination with trametinib suppresses refractory NRASQ61K melanoma. *Oncotarget* 2017, **8**(8)**:** 12576-12595.

11. Zhang F, Li Y, Zhang H, Huang E, Gao L, Luo W*, et al.* Anthelmintic mebendazole enhances cisplatin's effect on suppressing cell proliferation and promotes differentiation of head and neck squamous cell carcinoma (HNSCC). *Oncotarget* 2017, **8**(8)**:** 12968-12982.

12. Williamson T, Bai RY, Staedtke V, Huso D, Riggins GJ. Mebendazole and a non-steroidal anti-inflammatory combine to reduce tumor initiation in a colon cancer preclinical model. *Oncotarget* 2016, **7**(42)**:** 68571-68584.

13. Chen Q, Li Y, Zhou X, Li R. Oxibendazole inhibits prostate cancer cell growth. *Oncol Lett* 2018, **15**(2)**:** 2218-2226.

14. Nakajima A, Yamakuni T, Haraguchi M, Omae N, Song SY, Kato C*, et al.* Nobiletin, a citrus flavonoid that improves memory impairment, rescues bulbectomy-induced cholinergic neurodegeneration in mice. *J Pharmacol Sci* 2007, **105**(1)**:** 122-126.

15. Nakajima A, Yamakuni T, Matsuzaki K, Nakata N, Onozuka H, Yokosuka A*, et al.* Nobiletin, a citrus flavonoid, reverses learning impairment associated with N-methyl-D-aspartate receptor antagonism by activation of extracellular signal-regulated kinase signaling. *J Pharmacol Exp Ther* 2007, **321**(2)**:** 784-790.

16. Jiang YP, Guo H, Wang XB. Nobiletin (NOB) suppresses autophagic degradation via over-expressing AKT pathway and enhances apoptosis in multidrug-resistant SKOV3/TAX ovarian cancer cells. *Biomed Pharmacother* 2018, **103:** 29-37.

17. Chen J, Creed A, Chen AY, Huang H, Li Z, Rankin GO*, et al.* Nobiletin suppresses cell viability through AKT pathways in PC-3 and DU-145 prostate cancer cells. *BMC Pharmacol Toxicol* 2014, **15:** 59.
